# A Genome-Wide Association Scan on the Levels of Markers of Inflammation in Sardinians Reveals Associations That Underpin Its Complex Regulation

**DOI:** 10.1371/journal.pgen.1002480

**Published:** 2012-01-26

**Authors:** Silvia Naitza, Eleonora Porcu, Maristella Steri, Dennis D. Taub, Antonella Mulas, Xiang Xiao, James Strait, Mariano Dei, Sandra Lai, Fabio Busonero, Andrea Maschio, Gianluca Usala, Magdalena Zoledziewska, Carlo Sidore, Ilenia Zara, Maristella Pitzalis, Alessia Loi, Francesca Virdis, Roberta Piras, Francesca Deidda, Michael B. Whalen, Laura Crisponi, Antonio Concas, Carlo Podda, Sergio Uzzau, Paul Scheet, Dan L. Longo, Edward Lakatta, Gonçalo R. Abecasis, Antonio Cao, David Schlessinger, Manuela Uda, Serena Sanna, Francesco Cucca

**Affiliations:** 1 Istituto di Ricerca Genetica e Biomedica, Consiglio Nazionale delle Ricerche, Cagliari, Italy; 2 Intramural Research Program, National Institute on Aging, Baltimore, Maryland, United States of America; 3 University of Texas, MD Anderson Cancer Center, Department of Epidemiology, Houston, Texas, United States of America; 4 Dipartimento di Scienze Biomediche, Università di Sassari, Sassari, Italy; 5 Center for Statistical Genetics, Department of Biostatistics, University of Michigan, Ann Arbor, Michigan, United States of America; 6 Center for Advanced Studies, Research, and Development in Sardinia (CRS4), AGCT Program, Parco Scientifico e tecnologico della Sardegna, Pula, Italy; 7 High Performance Computing and Network, CRS4, Parco Tecnologico della Sardegna, Pula, Italy; 8 Porto Conte Ricerche, Località Tramariglio, Alghero, Sassari, Italy; FAS Center for Systems Biology, Harvard University, United States of America

## Abstract

Identifying the genes that influence levels of pro-inflammatory molecules can help to elucidate the mechanisms underlying this process. We first conducted a two-stage genome-wide association scan (GWAS) for the key inflammatory biomarkers Interleukin-6 (IL-6), the general measure of inflammation erythrocyte sedimentation rate (ESR), monocyte chemotactic protein-1 (MCP-1), and high-sensitivity C-reactive protein (hsCRP) in a large cohort of individuals from the founder population of Sardinia. By analysing 731,213 autosomal or X chromosome SNPs and an additional ∼1.9 million imputed variants in 4,694 individuals, we identified several SNPs associated with the selected quantitative trait loci (QTLs) and replicated all the top signals in an independent sample of 1,392 individuals from the same population. Next, to increase power to detect and resolve associations, we further genotyped the whole cohort (6,145 individuals) for 293,875 variants included on the ImmunoChip and MetaboChip custom arrays. Overall, our combined approach led to the identification of 9 genome-wide significant novel independent signals—5 of which were identified only with the custom arrays—and provided confirmatory evidence for an additional 7. Novel signals include: for IL-6, in the *ABO* gene (rs657152, p = 2.13×10^−29^); for ESR, at the *HBB* (rs4910472, p = 2.31×10^−11^) and *UCN119B*/*SPPL3* (rs11829037, p = 8.91×10^−10^) loci; for MCP-1, near its receptor *CCR2* (rs17141006, p = 7.53×10^−13^) and in *CADM3* (rs3026968, p = 7.63×10^−13^); for hsCRP, within the *CRP* gene (rs3093077, p = 5.73×10^−21^), near *DARC* (rs3845624, p = 1.43×10^−10^), *UNC119B*/*SPPL3* (rs11829037, p = 1.50×10^−14^), and *ICOSLG/AIRE* (rs113459440, p = 1.54×10^−08^) loci. Confirmatory evidence was found for IL-6 in the *IL-6R* gene (rs4129267); for ESR at *CR1* (rs12567990) and *TMEM57* (rs10903129); for MCP-1 at *DARC* (rs12075); and for hsCRP at *CRP* (rs1205), *HNF1A* (rs225918), and *APOC-I* (rs4420638). Our results improve the current knowledge of genetic variants underlying inflammation and provide novel clues for the understanding of the molecular mechanisms regulating this complex process.

## Author Summary

Inflammation is a protective response of our organism to harmful stimuli—such as germs, damaged cells, or irritants—and to initiate the healing process. It has also been implicated, with both protective and predisposing effects, in a number of different diseases; but many important details of this complex phenomenon are still unknown. Identifying the genes that influence levels of pro-inflammatory molecules can help to elucidate the factors and mechanisms underlying inflammation and their consequence on health. Genome-wide association scans (GWAS) have proved successful in revealing robust associations in both common diseases and quantitative traits. Here, we thus performed a multistage GWAS in a large cohort of individuals from Sardinia to examine the role of common genetic variants on the key inflammatory biomarkers Interleukin-6, erythrocyte sedimentation rate, monocyte chemotactic protein-1, and high-sensitivity C-reactive protein. Our work identified new genetic determinants associated with the quantitative levels of these inflammatory biomarkers and confirmed known ones. Overall, the data highlight an intricate regulation of this complex biological phenomenon and reveal proteins and mechanisms that can now be followed up with adequate functional studies.

## Introduction

Inflammation is a critical response to pathogens and injuries. Its control entails a coordinated cascade of biological events regulated by specific cells and molecular signals, in a complex process that is only partially understood. In this context, genetics can provide important clues, given that population studies indicate that about half of the inter-individual variability in biomarkers of inflammation is genetically determined and considering the achievements of GWA scans (GWAS) in complex trait analysis during the last few years [1]–[6]. To date, however, the genetic variants involved in the control of inflammation are still largely unidentified.

The relevance for clarifying the genetic bases of inflammation and understanding their mechanistic consequences is multi-fold. The immediate importance regards a better understanding of the regulation of the components of inflammation itself. Furthermore, recent population genetic studies have suggested that natural selection has shaped the evolution of innate immunity, with a specific pressure on those inflammatory genes that play a pivotal role in host-pathogen interactions [7], [8]. In addition, the inflammatory response can also influence in a positive or negative way the risk for several complex non-infectious diseases, as highlighted by recent studies on cardiopathologies and metastatic processes [9], [10]; knowing the variants involved in the process can thus have implications in different clinical settings.

To identify the genetic variants explaining the inter-individual variability in biomarkers of inflammation, we conducted a GWAS for the levels of the key inflammatory biomarkers interleukin-6 (IL-6), erythrocyte sedimentation rate (ESR), monocyte chemotactic protein-1 (MCP-1) and the C-reactive protein using the high-sensitivity assay (hsCRP) in a large cohort of Sardinian individuals from the SardiNIA study [11]. These markers represent different pathways and stages in the inflammatory cascade and their serum levels are used for the diagnosis and management of different inflammatory conditions during both the acute and chronic immune response.

## Results

We initially assessed 731,213 autosomal or X chromosome SNPs and imputed further ∼1.9 million variants in 4,694 individuals (Step 1) and then replicated the top signals in an additional 1,392 individuals (Step 2). To increase the detection power and provide useful information for the fine mapping stage we have also evaluated the whole cohort of 6,145 individuals with the ImmunoChip (151,085 variants) [12] and the MetaboChip (142,790 variants, of which 9,920 overlapped with ImmunoChip) [13] (Step 3). Overall we detected variants significantly associated with each of the traits assessed. The salient results are reported below.

### Step 1: Genome-Wide Association Scan

We identified several SNPs above the standard genome-wide significant threshold (5×10^−08^) in the SardiNIA discovery cohort (Table 1, Table S1 and Figure S1). The region surrounding each of these SNPs was studied in more detail as the respective traits were analyzed (see below).

**Table 1:** Top genome-wide association results for IL-6, ESR, MCP-1, and hsCRP.

				SardiNIA GWAS					SardiNIA stage 2				Combined	
Trait	Gene	Marker	Allele Minor/Major	N	RSQR	Freq	Effect^a^(SE)	p-value	N	Freq	Effect^a^(SE)	p-value	N	p-value
**IL-6**	***ABO***	**rs643434**	**A/G**	**4621**	**0.999**	**0.258**	**-0.245(0.026)**	**2.69×10^−21^**	**1390**	**0.264**	**−0.160(0.039)**	**4.07×10^−05^**	**6011**	**8.68×10^−25^**
ESR	*CR1*	rs12034598	A/G	4689	GEN	0.408	−0.143(0.022)	9.31×10^−11^	1392	0.370	−0.128(0.035)	2.19×10^−04^	6081	8.82×10^−14^
	***HBB***	**rs4910742**	**G/A**	**4689**	**GEN**	**0.066**	**−0.229(0.042)**	**6.34×10^−08^**	**1375**	**0.051**	**−0.199(0.076)**	**8.62×10^−03^**	**6064**	**1.89×10^−09^**
MCP-1	*DARC*	rs12075	A/G	4624	0.709	0.490	0.303(0.026)	1.68×10^−30^	1392	0.560	0.399(0.039)	4.93×10^−25^	6016	4.33×10^−51^
	***CADM3*** b	**rs3026968**	**T/C**	**4153**	**GEN**	**0.120**	**0.239(0.033)**	**7.63×10^−13^**	**1226**	**0.120**	**0.151(0.062)**	**1.47×10^−02^**	**5379**	**8.70×10^−14^**
hsCRP	*CRP*	rs1341665	A/G	4434	0.963	0.417	−0.195(0.024)	2.82×10^−16^	1069	0.371	−0.188(0.043)	1.32×10^−05^	5503	1.98×10^−20^
	***DARC*** b	**rs3845624**	**C/A**	**3985**	**GEN**	**0.470**	**0.140(0.220)**	**1.43×10^−10^**	**941**	**0.470**	**0.102(0.046)**	**2.66×10^−02^**	**4926**	**1.65×10^−11^**

The table summarizes top genome-wide association signals for IL-6, ESR, MCP-1 and hsCRP phenotypes in the HapMap based GWAS (Step 1), as well as results in the replication independent cohort (Step 2) and in the combined data-sets. For each marker, frequency and effect estimates are given with respect to the minor allele. Imputation quality scores (RSQ) are reported for imputed SNPs. Novel signals are indicated in bold.

a The effect size is measured in standard deviation units, being estimated as the β coefficient of the regression model when using the normalized trait (e.g. an effect size of 1.0 implies each additional copy of the allele being evaluated increases trait values by 1.0 standard deviations).

b Independent signals.

### IL-6

For IL-6, SNPs with p-values<5×10^−8^ were all located in the *ABO (a-1-3-N-acetylgalactosaminyltransferase*) locus on chromosome 9q34.1-q34.2 (Table S1), encoding the Histo-blood group ABO system transferase, with the strongest signal at rs643434 in intron 1 of the gene (p = 2.69×10^−21^, 0.69 pg/ml average increase per G allele) (Table 1). This SNP is in strong linkage disequilibrium (LD) with another associated variant, rs687289, which tags the O allele of the *ABO* locus (r^2^ = 0.931 in HapMap CEU).

### ESR

For ESR, we identified several associated SNPs on chromosome 1q32, all within the *CR1* (*complement component* (*3b/4b*) *receptor 1*) gene, a member of the receptors of the complement activation family, recently shown associated with ESR (Table S1) [14]. The strongest signal was observed at rs12034598 in intron 22 of *CR1*, with a p-value of 9.31×10^−11^ (1.024 mm/h average increase per G allele) (Table 1). This SNP is in strong LD with other associated variants including rs2274567 (r^2^ = 1), a non-synonymous SNP in exon 22 that causes a His1208Arg substitution predicted as potentially damaging by PolyPhen and affecting expression levels of CR1 on the erythrocytes (Table S1) [15], [16]. In the genomic region covered by *CR1* several copy number variations (CNVs) have been identified. However, none of the 38 SNPs in this region with p-value<5×10^−08^ (Table S1) tags the CNVs reported in a previous study [17], [18]. In addition, CNVs analysis with PennCNV [19] in individuals genotyped with the Affymetrix 6.0 microarray did not show presence of CNVs in our samples (unpublished data). Still, we could not exclude the presence of population-specific CNVs or common CNVs not directly interrogated by the Affymetrix probes.

We also found a locus suggestively associated with ESR on chromosomes 11p15 in the *β-globin locus control region* (*LCR*), which coordinates the expression of the globin genes (Table S1). The top signal was at marker rs4910742 (p = 6.34×10^−08^) (Table 1), which is a surrogate for the β^0^39 mutation carried by a large portion (11–13%) of the Sardinians and able to influence the levels of several blood indices, including number of RBCs [20], a parameter that has an inverse relationship with ESR. Accordingly, when we repeated the association analysis including in the model *β-Thalassemia* (*β-Thal*) carrier status as a covariate, the association at rs4910742 disappeared (p = 0.54 in SardiNIA).

### MCP-1

The GWAS results for MCP-1 levels revealed strong association signals on chromosome 1q22-q23 (Table S1). The associated region encompassed ∼500 kb and contained several genes, with the top signal detected in the *DARC* (*Duffy blood group chemokine receptor*) gene at marker rs12075 (p = 1.68×10^−30^, 36.78 pg/ml average increase per A allele), as also shown in a recent meta-analysis (Table 1) [21]. The association curve encompasses several other genes, including the *CADM3* (*cell adhesion molecule 3*) locus upstream of *DARC*, as well as in the *FCER1A* (*Fc fragment of IgE, high affinity I, receptor for alpha polypeptide*), *OR10J1* (*olfactory receptor, family 10, subfamily J, member 1*), and *OR10J5*, that have been previously reported to be associated with MCP-1 levels (Table S1) [22]. Interestingly, when we performed a conditional analysis on the top SNP in the *DARC* gene, SNP rs3026968 in the *CADM3* gene still showed a strong association (p = 4.26×10^−08^), indicating that this marker represents an independent signal (r^2^ with rs12075 = 0.043). SNPs with borderline association signals with MCP-1 levels were also found on chromosome 6p21.3, near the *HLA-DRB9* (*major histocompatibility complex, class II, DR beta 9*) pseudogene (rs9405112, p = 6.43×10^−08^); on chromosome 20q13, near the *CDH4* (*cadherin 4*) gene (rs6513566, p = 5.29×10^−08^), and on 3p21 at the 5′ of the *CCR2* gene (rs3918357, p = 8.49×10^−08^), encoding the chemokine (C-C motif) receptor 2, which acts as the MCP-1 receptor.

### hsCRP

For hsCRP, the strongest association signal was observed in the *CRP* (*C-reactive protein*) gene on chromosome 1q21-q23, confirming previous findings [23]–[25]. The top marker (rs1341665, p = 2.82×10^−16^, 0.692 mg/L average increase per G allele) is in strong LD with several variants, including rs1205, a 3-prime flanking region SNP previously implicated in *CRP* expression and systemic lupus erythematosus susceptibility (Table 1 and Table S1) [26]. In addition, we detected the presence of a novel independent signal at rs3845624 downstream of the *DARC* gene (p = 1.43×10^−10^, r^2^ = 0.015 with rs1341665 and r^2^ = 0.009 with rs1205). Indeed, when accounting for rs1341665, several SNPs in the *DARC* locus, and in particular rs3845624, still showed evidence for association (p = 4.75×10^−07^), suggesting a role for this gene in the regulation of CRP levels.

### Step 2: Follow-Up of Initial Findings

To corroborate our initial findings, we examined with TaqMan genotyping technology the 4 top associated SNPs (p<5×10^−08^), as well as 3 additional SNPs including the 2 independent signals in the *CADM3* and *DARC* loci (rs3026968 and rs3845624), and one suggestive SNP with p<10^−06^ near *HLA-DRB9* (rs9268858), in a group of 1,392 Sardinians enrolled in the same SardiNIA study but unrelated to the individuals analyzed in Step 1 GWAS. This independent cohort has been previously described as SardiNIA stage 2 [27]. Table 1 provides a summary of the follow-up results for the SNPs with the strongest association signal at each locus as well as a combined analysis.

Follow-up analysis of the top SNP rs643434 in *ABO* showed replication of this signal in SardiNIA stage 2 (p = 4.07×10^−05^, Table 1), supporting a role for this gene in regulating the levels of IL-6.

For ESR, replication was observed for both the top marker in the known *CR1* gene (rs12034598, p = 2.19×10^−04^ for its genotyped proxy rs650877 with r^2^ = 1), and the SNP in the β-globin *LCR*, (rs4910742, p = 8.62×10^−03^ for its genotyped proxy rs10500647 with r^2^ = 0.661) (see Table 1). As observed in the Step 1, when we repeated the association analysis for the latter SNP, including in the model *β-Thal* carrier status as a covariate, the association disappeared (p = 0.34).

The top SNP in the chemokine receptor gene *DARC* known to be associated with MCP-1 levels was also strongly replicated (rs12075, p = 4.93.×10^−25^). The relatively lower association signal showed by rs12075 in the SardiNIA discovery cohort (Step 1) compared to the follow-up cohort SardiNIA stage 2 is most likely due to the fact that it was imputed with a modest imputation score in the initial GWAS, whereas it was directly genotyped in the replication cohort. The association signal at rs3026968 in *CADM3* was also confirmed in SardiNIA stage 2 (p = 0.0147, Table 1), whereas the association at rs9405112 in the *HLA-DRB9* region was not (p = 0.59). Neither the signal in the *CDH4* gene, or that at rs3918357 in the *CCR2* gene were followed up; however, the latter supports a previously reported suggestive association in the *CCR2/CCR3* cytokine receptor gene cluster (rs12495098, r^2^ = 1 with rs3918357) [21].

Finally for hsCRP, the top SNP associated in the known *CRP* gene was fully confirmed (p = 1.32×10^−05^ for its perfect proxy rs2808628, r^2^ = 1) (Table 1), and replication was observed also for the independent signal at rs3845624, near *DARC* (p = 0.027, Table 1).

### Step 3: Gene-Specific Scan using Custom Genotyping Arrays

**Figure 1:**
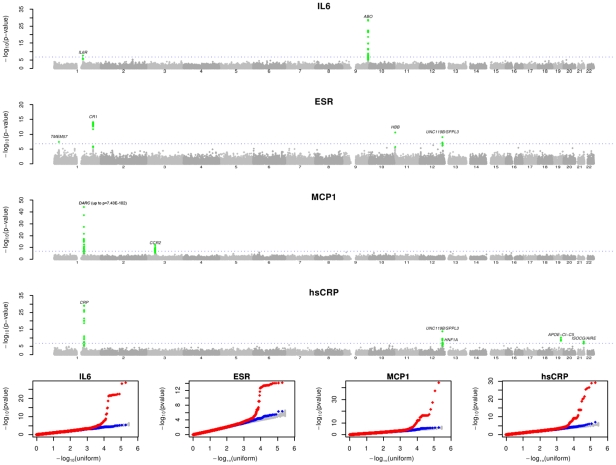
Manhattan plot and QQ plot of association findings. The figure summarizes the association results obtained on the ImmunoChip and MetaboChip markers (Step 3). The blue dotted line marks the Bonferroni threshold significance levels (1.7×10^−7^), and SNPs in loci exceeding this threshold are highlighted in green. The bottom panel represents the QQ plot, where the red line corresponds to all test statistics, and the blue line to results after excluding statistics at top markers (highlighted in green in the Manhattan Plot). The gray area corresponds to the 90% confidence region from a null distribution of P values (generated from 100 simulations).

**Figure 2:**
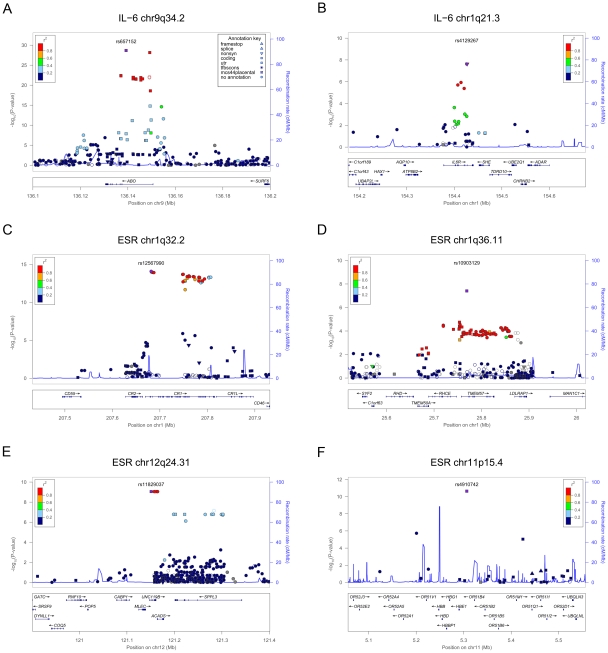
Zoom views of the association results in the loci associated with IL-6 and ESR. Each panel shows the association curve around the strongest SNP, which is highlighted with a purple dot. The SNPs are coloured according to their linkage disequilibrium (r^2^) with the top variant in the 1000 Genomes European data set, with symbols that reflect genomic annotation as indicated in the legend. Arrows highlight independent signals, if any, described in the manuscript; while light blue lines indicate the recombination rate, according to the right-hand Y axis. Genomic positions are as in build 37. Gene transcripts are annotated in the lower box. Plots were drawn using the standalone LocusZoom version [65].

**Figure 3:**
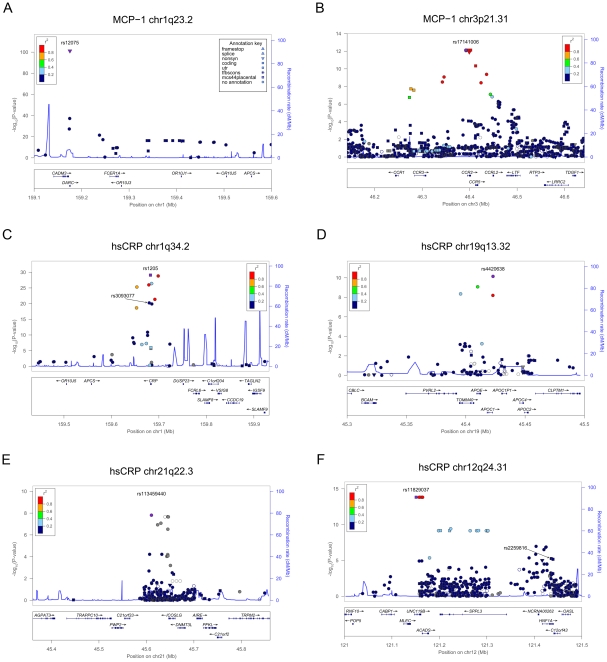
Zoom views of the association results in the loci associated with MCP-1 and hsCRP. Each panel shows the association curve around the strongest SNP, which is highlighted with a purple dot. The SNPs are coloured according to their linkage disequilibrium (r^2^) with the top variant in the 1000 Genomes European data set, with symbols that reflect genomic annotation as indicated in the legend. Arrows highlight independent signals, if any, described in the manuscript; while light blue lines indicate the recombination rate, according to the right-hand Y axis. Genomic positions are as in build 37. Gene transcripts are annotated in the lower box. Plots were drawn using the standalone LocusZoom version [65].

To refine the contribution of the detected loci and increase the power to detect novel signals, we performed an additional association scan by testing 293,875 variants assessed in the whole SardiNIA cohort (6,145 individuals, including the discovery and follow-up cohorts from Steps 1 and 2) by genotyping with the ImmunoChip [12] and the MetaboChip [13], two Illumina custom arrays designed to follow up regions of prior interest in immune- and metabolic-related traits and diseases, respectively, as detailed in the Methods section. With this approach, we not only validated with an independent genotyping method, and refined all the association results at the previously described loci (see Figure S1, Table 1), but also identified novel signals for all traits (Figure 1, Table 2). A detailed view of the associated regions is illustrated in Figure 2 and Figure 3, and results discussed below. The effect of the associated variants on trait variability per genotype is represented in Figure S2 and Figure S3.

**Table 2:** Top association signals for IL-6, ESR, MCP-1, and hsCRP in the ImmunoChip and MetaboChip data-sets.

Trait	Gene	Marker	Allele Minor/ Major	N	Freq	Effect^a^(SE)	p-value	Array^b^	r^2^ with GWAS (SNP)
IL-6	***ABO***	**rs657152**	**T/G**	**5915**	**0.269**	**−0.219 (0.019)**	**2.13×10^−29^**	**I**	**1 (rs643434)**
	*IL6R*	rs4129267	T/C	5915	0.260	0.109 (0.020)	2.36×10^−08^	I	–
ESR	*CR1*	rs12567990	C/T	6021	0.408	−0.152 (0.020)	8.26×10^−15^	I	0.945 (rs12034598)
	***HBB***	**rs4910742**	**G/A**	**6021**	**0.075**	**−0.208 (0.031)**	**2.31×10^−11^**	**I**	**(same SNP)**
	*TMEM57*	rs10903129	G/A	6021	0.339	−0.093 (0.017)	3.91×10^−08^	I	–
	***UNC119B/SPPL3***	**rs11829037**	**T/C**	**6106**	**0.009**	**0.523 (0.085)**	**8.91×10^−10^**	**M**	**–**
MCP-1	*DARC*	rs12075	G/A	6010	0.445	−0.405 (0.019)	7.43×10^−102^	M	(same SNP)
	***CCR2***	**rs17141006**	**C/T**	**5924**	**0.101**	**0.253 (0.035)**	**7.53×10^−13^**	**I**	**0.997 (rs3918357)**
hsCRP	*CRP*	rs1205	T/C	5705	0.383	−0.209 (0.018)	8.20×10^−30^	I	0.961 (rs1341665)
	***CRP*** c *^,^* d	**rs3093077**	**G/T**	**5705**	**0.118**	**0.173 (0.027)**	**5.73×10^−21^**	**I**	**0.043 (rs3845624)**
	***UNC119B/SPPL3***	**rs11829037**	**T/C**	**5791**	**0.009**	**0.713 (0.093)**	**1.50×10^−14^**	**M**	**–**
	*HNF1A* c *^,^* d	rs2259816	A/C	5703	0.335	−0.114 (0.019)	5.41×10^−06^	I	–
	*APOC-I*	rs4420638	G/A	5657	0.094	−0.200 (0.031)	7.13×10^−11^	I	–
	***ISOCG/AIRE***	**rs113459440**	**T/C**	**5704**	**0.003**	**0.819 (0.145)**	**1.54×10^−08^**	**I**	**–**

The table summarizes top association signals for IL-6, ESR, MCP-1 and hsCRP phenotypes in the ImmunoChip and MetaboChip data-sets (Step 3). For each marker, frequency and effect estimates are given with respect to the minor allele. We also reported the r^2^ with the SNP detected in the GWAS scan (Step 1). Novel signals are indicated in bold.

a The effect size is measured in standard deviation units, being estimated as the β coefficient of the regression model when using the normalized trait (e.g. an effect size of 1.0 implies each additional copy of the allele being evaluated increases trait values by 1.0 standard deviations).

b I =  ImmunoChip, M =  MetaboChip.

c The table reports the pvalue on the primary analysis. On the conditional analysis, the pvalue for the independent SNPs were: rs12378220, 9.43×10^−08^; rs3093077, 9.02×10^−11^; rs2259816, 7.58×10^−10^.

d Independent signals.

### IL-6

For IL-6 levels, besides corroborating the association at the *ABO* gene (strongest hit rs657152, p = 2.13×10^−29^), we also observed a signal at rs4129267 (p = 2.36×10^−08^, with an average increase of 0.220 pg/ml per T allele), in the *IL-6R* (*IL-6 receptor*) gene (Table 2, Figure 2A and 2B, and Figure S2). This SNP is a proxy of the functional SNP rs8192284 (r^2^ = 0.982 in HapMap CEU) affecting cleavage of IL-6 soluble receptor (IL-6 sR), which was previously found associated with both IL-6 sR and IL-6 levels by admixture mapping and candidate gene analysis in African and European Americans [28]. SNP rs4129267 was genotyped in the Step 1 GWAS but observed with a lower p-value (p = 2.45×10^−04^). Conditional analysis did not reveal any independent signals at such loci. The top variants at *ABO* (rs657152) and *IL-6R* (rs4129267) explain 2.2% of the total phenotype variation.

### ESR

The scan for ESR, in addition to strong confirmatory signals in *CR1* (strongest hit rs12567990, p = 8.26×10^−15^), detected a novel associated SNP, rs10903129, in *TMEM57* (*Transmembrane protein 57*)(p = 3.91×10^−08^, with an average increase of 0.581 mm/h per G allele) (Table 2, Figure 2C and 2D, and Figure S2). SNP rs10903129 was analysed in the Step 1 GWAS, but its p-value did not reach genome-wide significance (p = 9.30×10^−05^) and thus it was not considered for follow-up in Step 2. Although this gene encodes a largely uncharacterized protein, polymorphisms in the region have been previously reported associated with lipid levels, CHD and more recently with ESR [14], [29]. We also detected a novel signal on chromosome 12q24.31 near the *UNC119B* (*Unc-119 homolog B*) and *SPPL3* (*Signal peptide peptidase-like 3*) genes at a low frequency SNP, rs11829037, with a large effect (p = 8.91×10^−10^, MAF =  0.009, average increase of 4.657 mm/h per the minor T allele) (Figure 2E and Table 2). It was genotyped by the MetaboChip, but the association at this locus was supported by SNPs genotyped with both arrays, and by more common SNPs (MAF up to 0.05 for the 18 SNPs with p-value<10^−6^). It is missing and not well tagged in the HapMap data set, which provides an explanation as to why it was not discovered in the initial scan (step 1). Finally, we confirmed the association at rs4910742 (p = 2.31×10^−11^) in the *HBB* locus (Figure 2F). Conditional analysis did not reveal any independent signals at such loci. The top variants at *CR1* (rs12567990), *HBB* (rs4910742), *TMEM57* (rs10903129) and *UNC119B/SPPL3* (rs11829037) explain 2.3% of the total trait variation.

### MCP-1

For MCP-1, the association with the coding SNP in *DARC* was corroborated with a striking p-value (rs12075, p =  7.43×10^−102^) (Figure 3A and Table 2). In addition, SNP rs17141006, 10 kb upstream of its receptor *CCR2*, correlated with the previous borderline signal (r^2^ = 0.997), reached genome-wide significance (rs17141006, p = 7.53×10^−13^, average increase per C allele was 42.14 pg/ml) (Table 2, Figure 3B and Figure S2). As mentioned earlier, SNPs in the *CCR2/CCR3* receptor cluster associated with MCP-1 levels were previously reported by Shnabnel et al. [21], although these associations did not reach the genome-wide significance threshold. Our study thus refines the association and points to *CCR2* as the most likely candidate for a role in the levels of MCP-1. We also carried out a search for independent SNPs by conditioning on the strongest associated variant, but this analysis did not reveal any evidence. The independent signal in *CADM3* or an adequate proxy were not included on the custom arrays, and thus it could not be tested in this data set. However, since the SNP was available in both the SardiNIA discovery cohort (Step 1) and SardiNIA stage 2 data sets, genotypes were accessible for the entire cohort and independency was confirmed. We estimated that all together the top variants at *DARC* (rs12075), *CCR2* (rs17141006), and *CADM3* (rs3845624) explain 9.8% of the phenotypic variation.

### hsCRP

For hsCRP, strong association signals were detected in the previously described *CRP* gene (rs1205, p = 8.20×10^−30^) and in the 3′ of *APOC-I* (*Apolipoprotein C-I*) gene (rs4420638, p = 7.12×10^−11^) (Figure 3C and 3D and Table 2), a well known determinant of serum hsCRP that did not reach statistical significance (p = 2.85×10^−5^) in our initial scan (Stage 1) [25]. A novel and previously unknown signal with a large phenotypic impact was identified at a rare variant, rs113459440 (p = 1.54×10^−08^, MAF = 0.003, average increase of 5.35 mg/L per T allele), near *ICOSLG* (*Inducible T-cell co-stimulator ligand*) and the *AIRE* (Autoimmune regulator) genes (Figure 3E and Figure S3). Common variants at the first gene have been associated by GWAS with the risk of Ulcerative colitis, Celiac disease, Chron's disease and ankylosin spondilytis in Europeans [30]–[32], whereas at the latter gene with Rheumatoid arthritis in Japanese [33]. The *AIRE* gene is also responsible for Autoimmune polyendocrinopathy syndrome, type I (APECED), an autosomal recessive autoimmune disease relatively common in Sardinia (OMIM # 607358). Association was supported by other SNPs genotyped with the ImmunoChip (7 SNPs with p-value<10^−6^, with MAF up to 0.007). In addition, we observed that the same low frequency SNP at *UNC119B/SPPL3* associated with ESR levels was also associated with hsCRP (rs11829037, p = 1.50×10^−14^, average increase of 3.68 mg/L per T allele) (Figure 3F, Table 2 and Figure S3). Similarly to ESR, association is likely to be genuine, supported by SNPs genotyped with both arrays and several common SNPs (MAF up to 0.38 for the 22 SNPs with p-value<10^−6^). Notably, signals at SNPs within *SSLP3* were previously detected associated with CRP levels in an isolated founder population from the Pacific Island of Kosrae, although the p-values did not reach the genome-wide threshold [34]. None of those SNPs was correlated to the top SNP associated in our study; however the four SNPs which were genotyped (rs10437838, rs6489780, rs1039302, rs10431387) showed consistent direction of allele effects (increasing value for the minor allele) as in Lowe et al., albeit with weak evidence (0.04<p<0.09). This further indicates that association at this locus cannot be spurious. Conditional analysis revealed the presence of two independent signals. The first was within the *CRP* gene, at SNP rs3093077 (p = 9.02×10^−11^ after conditioning for rs1205, with average increase of 0.724 mg/L per G allele) (Figure 3C, Table 2 and Figure S3). This marker is independent from the signal observed 461 Kb downstream in the Step 1 GWAS scan, rs3845624, near the *DARC* gene (r^2^ = 0.043). Indeed, when accounting for rs1205 and rs3093077 in the HapMap-based GWAS data set, the association signal at rs3845624 was still significant. The second independent signal was at rs2259816 (p = 7.58×10^−10^ after conditioning for rs11829037, with average increase of 0.381 mg/L per C allele), in an intron of the *HNF1A* (*Hepatic nuclear factor-1α*) gene about 300 Kb downstream from the *UNC119B/SPPL3* locus (Figure 3F, Table 2, and Figure S3). This marker is a perfect proxy of rs1169310, a variant reported by a previous study [24]. The best signal at this locus on our initial GWAS (Step 1) was at a linked SNP, rs7953249 (r2 = 0.5), which did not reach genome-wide significance level (p = 7×10^−06^). Overall the top variants at *CRP* (rs1205), *APOC-I* (rs4420638), *ICOSLG/AIRE* (rs113459440), *UNC119B/SPPL3* (rs11829037), and the independent variants at *CRP* (rs3093077), *DARC* (rs3845624) and *HNF1A* (rs2259816) explain 5.6% of the phenotypic variation of this trait.

## Discussion

Our results, besides confirming previous associations, highlight new determinants for variation at the major inflammatory biomarkers IL-6, ESR, MCP-1 and hsCRP.

Specifically, we found a novel highly significant association between IL-6 expression levels and the *ABO* locus, with our top associated marker tagging the O allele. This association is of special interest, given the numerous biological effects of this cytokine as well as the associations previously reported of the O allele with both inflammatory traits and diseases [35]–[38]. In contrast to individuals with A and B alleles, individuals with the O blood group do not produce either the A or B antigens because of a single-base deletion in the gene sequence, whose product catalyzes the transfer of carbohydrates to the H antigen, forming the antigenic structure of the ABO blood group. Our data show that individuals carrying two copies of the G allele at our top SNP, corresponding to blood type O carriers, display highly increased IL-6 circulating levels compared to non-O carriers (average increase of 1.38 pg/ml for homozygotes of the G allele, compared to opposite homozygotes). This is consistent with the observation that blood type O individuals show an enhanced inflammatory response to *Helicobacter pylori*, with a significantly higher release of IL-6 [39]. The detected association at the *ABO* locus with the IL-6 phenotype may also provide a mechanistic clue for previous associations of the O blood group with various diseases with an inflammatory component such as cancer and heart disease, although determining the workings of this puzzle will likely also require specific functional studies.

Our study also revealed novel associations with ESR levels at the *HBB* and the *UNC119B/SPPL3* loci. Although the impact of the associated variants at *HBB* in ESR values is somewhat expected in Sardinia because of the high frequency of carriers of β-Thalassemia (see Results), our work indicates a direct link supported by a genetic association. The link of *UNC119B/*SPPL3 with ESR is currently less clear. Interestingly, we also found that the same *UNC119B/SPPL3* variant was associated with hsCRP levels, a finding supported by a recent study showing suggestive evidence at SNPs within the *SPPL3* gene with CRP levels variation [34]. As expected by the strong correlation of CRP blood circulating levels and ESR (i.e., high blood levels of acute phase proteins increase ESR), the same allele of the associated SNP at *UNC119B/SPPL3* increases both CRP and ESR, further supporting that the association at this locus is genuine.

Our results highlighted a novel association at the MCP-1 receptor *CCR2*, with a clear involvement with MCP-1 levels, previously only suggestively associated with this trait [21]. Confirming previous findings of Schabnel and colleagues [21], we also found robust evidence of association between MCP-1 and SNPs in the *DARC* gene, an unusual transmembrane chemokine receptor, which binds the two main families of inflammatory chemokines, CXC and CC (i.e., MCP-1). The top signal (rs12075) is a non-synonymous SNP located in exon 3 of the gene, that generates a Gly42Asp amino acid change in the DARC protein. The predicted impact of the mutation, as well as the strength of the association signal compared to all nearby variants, suggests that it represents a causal variant, as previously hypothesized [21]. However and intriguingly, our results indicate that the association in the region is complex, with one novel genome-wide significant independent signal at the upstream gene *CADM3*. In addition, we also observed that SNPs near the *DARC* gene are associated with variation in CRP levels. Although the biological implications of these SNPs on DARC function are at present unclear, this is consistent with the observation that MCP-1 production by endothelial cells rises in response to CRP [40]. The *DARC* associations with CRP and MCP-1 were genetically independent of each other, supporting the notion of a complex correlation between hsCRP and MCP-1, and suggesting a multi-layered control of expression of the inflammation response in the *DARC* region.

Finally, in spite of the small sample size compared with the large meta-analyses conducted so far [25], our study identified several new variants associated with hsCRP levels, including an independent signal at *CRP*, the signal at the previously discussed *UNC119B/SPPL3* locus and an unexpected signal at *ISOCG/AIRE*. Although the SNP associated with hsCRP at the *UNC119B/SPPL3* locus is independent and not correlated (r2<0.1) with the known signal at *HNF1A* located about 300 kb downstream [24], at present we cannot exclude that this SNP may act as an eQTL or more generally in the regulation of *HNF1A* expression.

Interestingly, the majority of the association signals (and specifically at *IL6R*, *TMEM57*, *UNC119B/SPPL3*, *CCR2*, *APOC-I*, *HNF1A*, the *CRP* independent signal, and *ICOCG/AIRE*), were observed, at least at the genome-wide level of significance, only after genotyping the MetaboChip and ImmunoChip custom arrays, which were typed in our cohort primarily to assess other phenotypes. All these signals, apart from that at *ICOCG/AIRE*, had supporting evidence for the involvement in the specific trait variation from previous reports, indicating that the associations are not spurious. The strongest variants were either not genotyped with the commercial arrays used in our initial scan, missing or poorly tagged in the HapMap-based reference panel we used for imputation, or only partially genotyped (given our genotyping strategy), resulting in inadequate power for being detected at the required significance level in the GWAS scan. This suggests that cost effective custom arrays could improve our understanding of the genetics underlying trait variation even for a phenotype, such as inflammation, for which the array was not specifically designed.

Understanding the effects of the protein products of all the discovered loci in inflammation is an important goal, which may also likely have clinical implications. For instance, whereas CRP and ESR are the most widely used non-specific diagnostic markers of inflammation, the factors and fine mechanisms regulating their levels and interfering with them are only partially understood.

Overall, our results contribute to improve the current knowledge of the regulation of the inflammatory response. While inflammation is canonically thought of as involving leukocyte migration and infiltration, the fact that several of the variants identified are better noted in erythrocyte function may suggest a more active role for the red cell in this process, beyond its obligate role in ESR. Notably, four of the associated loci (*ABO*, *HBB*, *DARC* and *CR1*) have been implicated in resistance to malaria, a disease endemic in Sardinia until a few decades ago [41]–[45]. This raises the possibility that the genetic selection imposed by malaria may have contributed to shaping levels of inflammation, at least for these specific inflammatory biomarkers, in this population [46]. Still, a link between these specific genes and variants with malaria remains speculative and needs to be further assessed with adequate biological and genetic analyses; for instance, they could be tested and cross-compared with future statistically well powered GWAS on Malaria and other infectious disorders.

A related potential detrimental consequence of inflammation is that polymorphisms which have been selected because they promote pro-inflammatory responses may increase the risk for diseases with an inflammatory component [47], [48], particularly those that show a high frequency in Sardinia, such as Multiple Sclerosis (MS) and Type 1 diabetes (T1D) [49], [50]. However, we could not find any evidence of association of the top SNPs associated with pro-inflammatory markers in a sample-set of 2,280 MS cases, 1,377 T1D cases and 1,922 unrelated controls, all from Sardinia [51], with a power of 60% and 33% to detect variants with an odds ratio of 1.4 and MAF of 0.1 at a significance level of 1×10^−07^, indicating that larger sample sizes are required to identify association at variants with smaller effects or of lower frequency (data not shown). Similarly, these variants were not found associated to other autoimmune diseases in larger data-sets (T1Dbase, http://t1dbase.org; and the GWAS Catalog, http://www.genome.gov/gwastudies/).

Another possibility is that these pro-inflammatory variants play a positive role in protection against serious diseases. For instance, the *ABO O* allele is also associated with a reduced risk of myocardial infarction and pancreatic and skin cancer [52]–[54]. Our results suggest that an increase in the circulating levels of IL-6 can indeed contribute to these associations involving the O group.

In conclusion, our work highlights important aspects of the complex and multilayered regulation of inflammation and may provide a route to understanding possible attendant effects on a number of serious diseases.

## Methods

### Ethics Statement

All individuals studied and all analyses on their samples were done according to the Declaration of Helsinki and informed consents were approved by the local ethics committee for the Istituto di Ricerca Genetica e Biomedica-CNR (IRGB-CNR; Cagliari, Italy) and by MedStar Research Institute, responsible for intramural research at the National Institutes of Aging, Baltimore, Maryland, United States.

### Sample Description

We recruited and phenotyped 6,148 individuals, males and females, ages 14–102 y, from a cluster of four towns in the Lanusei Valley of Sardinia [11]. During physical examination, a blood sample was collected from each individual and divided into two aliquots. One aliquot was used for DNA extraction and the other to characterize several blood phenotypes, including evaluation of serum levels of hsCRP, IL-6, MCP-1, and values of ESR. Descriptive statistics of the study cohort are shown in Table S2. Serum levels of hsCRP were measured by the high sensitivity Vermont assay (University of Vermont, Burlington), an enzyme-linked immunosorbent assay calibrated with WHO Reference Material [55]. The lower detection limit of this assay is 0.007 mg/l, with an inter-assay coefficient of variation of 5.14%. Serum levels of IL-6 and MCP-1 were measured by Quantikine High Sensitive Human Immunoassays (R&D Systems, Inc.), according to manufacturer's instructions. This method employs solid-phase ELISA techniques. For IL-6, the lower detection limit is 0.039 pg/ml. The intra-assay coefficient of variations (CVs) were 6.9% to 7.8% over the range 0.43–5.53 pg/ml. For MCP-1, the lower detection limit is 5.0 pg/ml. The intra-assay coefficient of variations (CVs) were 4.7% to 7.8% over the range 76.7–1121 pg/ml. ESR was measured using sedimentation measurement tubes buffered with 3.8% sodium citrate (Venoject-Terumo). After mixing of 2.4 ml of blood with the additive, tubes were left in a vertical position in the specific support with graduation markings for 30 minutes to allow sedimentation of the erythrocytes by gravity. The erythrocyte sedimentation rate is calculated in Westergreen units (mm/h) determining the length at the plasma/erythrocyte cell interface level within the sedimentation tube. Samples affected by multiple sclerosis (MS) and type 1 diabetes (T1D) used for the side case-control analysis briefly reported in the Discussion were recruited from all the island as previously described (51). Only 20 of these samples overlapped with those in the SardiNIA cohort.

### GWAS Genotyping and Statistical Analysis

During the study, we genotyped 4,694 individuals selected from the whole sample to represent the largest available families, regardless of their phenotypic values. Specifically, 1,412 were genotyped with the 500 K Affymetrix Mapping Array set, 3,329 with the 10 K Mapping Array set, with 436 individuals genotyped with both arrays. We also recently typed 1,097 individuals with the Affymetrix 6.0 chip, of which 1,004 and 66 were also typed with the 10 K and 500 K chips respectively. This genotyping strategy allowed us to examine the majority of our cohort in a cost-effective manner since genotypes for the SNPs that passed quality control checks could be propagated through the pedigree using imputation. Measurements of inflammatory biomarkers were available for 4,137, 4,292, 4,295 and 3,596 individuals for hsCRP, IL-6, MCP-1 and ESR, respectively, among the 4,694 genotyped. A total of 731,209 autosomal SNPs passed stringent quality control checks. Quality checks for the 10 K and 500 K chips were described previously [56]. For the Affymetrix 6.0 chip, similar criteria were used, as detailed in Table S3. In addition, we also removed SNPs in common between the other chips that showed an high level of discordance or that generated too many discrepancies when comparing genotypes across 11 duplicates. After performing quality control checks and merging genotypes from the three gene chip platforms, we used the quality controlled 731,209 autosomal markers to estimate genotypes for all polymorphic SNPs in the CEU HapMap population (release 22) [57], in the individuals genotyped with the 500 K Array and the 6.0 Affymetrix chip separately using the MaCH software [58]. Taking advantage of the relatedness among individuals in the SardiNIA sample, we carried out a second round of computational analysis to impute genotypes at all SNPs in the individuals who were genotyped only with the Affymetrix Mapping 10 K Array, being mostly offspring and siblings of the individuals genotyped at high density. At this second round of imputation, we focused on the SNPs for which the imputation procedure predicted r^2^>0.30 between true and imputed genotypes and for which the inferred genotype did not generate an excess of Mendelian errors. We then used a modified version of the Lander-Green algorithm, as previously described [3], [56] to estimate IBD sharing at the location of the SNPs being tested and identify stretches of haplotype shared with close relatives who were genotyped at higher density and probabilistically infer missing genotypes. The within-family imputation procedure and the association test are implemented in Merlin software [59], [60]. Due to computational constraints, we divided large pedigrees into sub-units with “bit-complexity” of 21 or less (typically, 25–30 individuals) before analysis.

For association, we evaluated the additive effect of genotyped and imputed SNPs on inflammatory biomarker levels using a family-based association test implemented in Merlin (–fastassoc option) [59], [60]. This test accounts for relatedness under the assumption that the samples analyzed are from an ethnically homogeneous population [59], [60], and this is suggested by demographic records indicating that 89% of the participants were born in the same 31 Km^2^ area and for 95% of the volunteers, both parents and all grandparents were born in Sardinia [11]. At each SNP, levels of each biomarker of inflammation were regressed onto allele counts in a regression model that included gender, age, and age-squared as covariates. We also used a second model, which included body-mass index (BMI) and smoking status as additional covariates, as these have been previously implicated as being associated with inflammatory biomarker levels [24]. Here we report the results from the second model, where the inclusion of the additional covariates improved the variance explained by the model (from 1.8% to 3.7% for CRP, from 18.1% to 19.5% for ESR, from 6.8% to 7.6% for IL-6, and from 3% to 3.3% for MCP-1). Genomic control parameters show negligible inflation (1.049, 1.039, 1.031 and 1.115, respectively for hsCRP, MCP-1, IL-6 and ESR); nevertheless the corresponding correction factors were applied to the GWAS results to completely avoid spurious associations.

### Follow-Up Sample

The SardiNIA stage 2 cohort was used to follow-up initial findings [27]. Genotyping of specific SNPs was performed in Sardinian individuals selected for replication efforts using TaqMan single SNP genotyping assays (Applied Biosystems). In particular, we genotyped and analysed 1,392 individuals from the SardiNIA stage 2 cohort, who were unrelated (kinship coefficient = 0) to the individuals analysed in the GWAS.

### ImmunoChip and MetaboChip: Genotyping and Statistical Analysis

We successfully genotyped 6,145 samples using the MetaboChip and ImmunoChip arrays (Illumina). The MetaboChip was designed in collaboration with several international consortia [3], [61], [62] with the aim to fine map association loci detected through GWAS for a variety of traits. Part of the design included a set of wild-card SNPs chosen by individual research groups; the SardiNIA study promoted several SNPs associated with a wide range of traits, including rs12075. The ImmunoChip is also a consortium based array, designed to fine map loci associated to 12 immunologically related human diseases, or immune-mediated disease loci, as well as a set of wild-card SNPs. The SardiNIA study had not role in the design of this array, and a full detailed description is provided elsewhere [12]. All samples had a genotyping call rate >98%, and SNP genotypes were carefully assessed though several quality control checks. In particular, we removed markers with call rate <98%, with strong deviation from HWE (p<10^−6^), that were monomorphic or leading to an excess of Mendelian errors (defined as >1% of the families). A detailed breakdown of markers excluded by each filter criteria is provided on Table S4. Since the majority of the variants included on these custom arrays are of low frequency compared to the GWAS data set (average MAF =  0.176, compared to 0.219 observed in GWAS), the impact of hidden population structure and imprecise modelling of relatedness due to pedigree splitting (task we performed on the GWAS data set due to computational constraints) could be problematic. Analysis was thus carried out using EMMAX, a variant component model that overcomes such issues by using a genomic-based kinship matrix [63]. To calculate the kinship matrix, we used all SNPs that passed quality control checks but excluding those with MAF between 0 and 1%. Association analysis was subsequently performed testing all QCed SNPs (Table S4), in spite of their minor allele frequency. Observed genomic lambda were 1.01, 0.962, 1.00 and 1.01, respectively for IL6, VES, MCP1, and hsCRP (as a note, genomic lambda using Merlin on the same data set were 1.41, 1, 1.26 and 1.14, respectively). To declare an association significant, we used a Bonferroni threshold of 0.05/293,875 = 1.7×10^−7^.

### Variance Explained

The variance explained by the strongest associated SNPs was calculated, for each trait, as the difference of R2 adjusted observed in the full and the basic models, where the full model contains all the independent SNPs in addition to the covariates.

### Conditional Analysis

We performed conditional analysis at each locus by adding the top associated SNP to the already included covariates, and testing for association the remaining SNPs at the locus. A marker was declared independent only if the p-value observed in the conditional analysis reached genome-wide significance threshold (5×10^−08^ in the Step1 GWAS, and 1.7×10^−07^ in the custom-array based dataset) [64].

## Supporting Information

Figure S1 Manhattan plot and QQ plot of association findings in step 1 GWAS. The figure summarizes the genome-wide association scan results combined across the data-sets by inverse variance weighting. The blue dotted line marks the threshold for genome-wide significance (5×10^−08^) [64]. SNPs in loci exceeding this threshold are highlighted in green. The bottom panel represents the QQ plot, where the red line corresponds to all test statistics, and the blue line to results after excluding statistics at top markers (highlighted in green in the Manhattan Plot). The gray area corresponds to the 90% confidence region from a null distribution of P values (generated from 100 simulations).
(PNG)

Figure S2 Boxplots for levels of IL-6, ESR, and MCP-1 for each genotype at the top associated SNPs. Standard boxplots are drawn with min, 0.25 quantile; median, 0.75 quantile; and 0.75 quantile+ 1.5*IQR for the levels of unadjusted biomarkers IL-6 (pg/ml), ESR (mm/h), and MCP-1 (pg/ml) in the original units. For each boxplot the name of the biomarker and the associated SNP are indicated, as well as the number of individuals per genotype.
(PNG)

Figure S3 Boxplots for levels of hsCRP for each genotype at the top associated SNPs. Standard boxplots are drawn with min, 0.25 quantile; median, 0.75 quantile; and 0.75 quantile+ 1.5*IQR for the levels of unadjusted hs-CRP (mg/ml) biomarker in the original units. For each boxplot the name of the biomarker and the associated SNP are indicated, as well as the number of individuals per genotype.
(PNG)

Table S1 Association results for SNPs with p-value<10^−5^ for inflammatory biomarkers in the step 1 GWAS. The table summarizes the association results at SNPs with p-value<10^−5^ for each inflammatory biomarkers. The effect size is measured in standard deviations (e.g. an effect size of 1.0 implies each additional copy of the allele being evaluated increases trait values by 1.0 standard deviations) and it refers to allele 1. The r^2^ between each imputed genotype and the true underlying genotype is provided (RSQ) and serves as a quality-control metric. The percentage of the variance explained by each markers is also reported (H2), and the column “I/G” indicates whether the SNP has been imputed or genotyped. Physical positions are given according to build 36. SNPs above genome-wide threshold (p<5×10^−8^) are indicated in bold. EA, effect allele; OA, other allele.
(DOC)

Table S2 Descriptive statistics for the SardiNIA cohort. The table shows the basic clinical characteristics of the SardiNIA samples.
(DOCX)

Table S3 Quality Control data for Affymetrix 6.0 chips and imputation.
(DOCX)

Table S4 Quality Controls for the MetaboChip and ImmunoChip data-sets. The table shows the breakdown of each criteria applied and the relative number of markers removed. The same marker could have failed one or more criteria. **a** excess was defined as >1% of the families. **b** SNPs on chromosomes X and Y were discarded for the analysis with EMMAX.
(DOCX)
