# Supplementary figures and images for: A Genome-Wide Association Scan on the Levels of Markers of Inflammation in Sardinians Reveals Associations That Underpin Its Complex Regulation
Source: PLoS Genet. 2012 Jan 26;8(1):e1002480. doi: 10.1371/journal.pgen.1002480 (PMC3266885; doi:10.1371/journal.pgen.1002480)

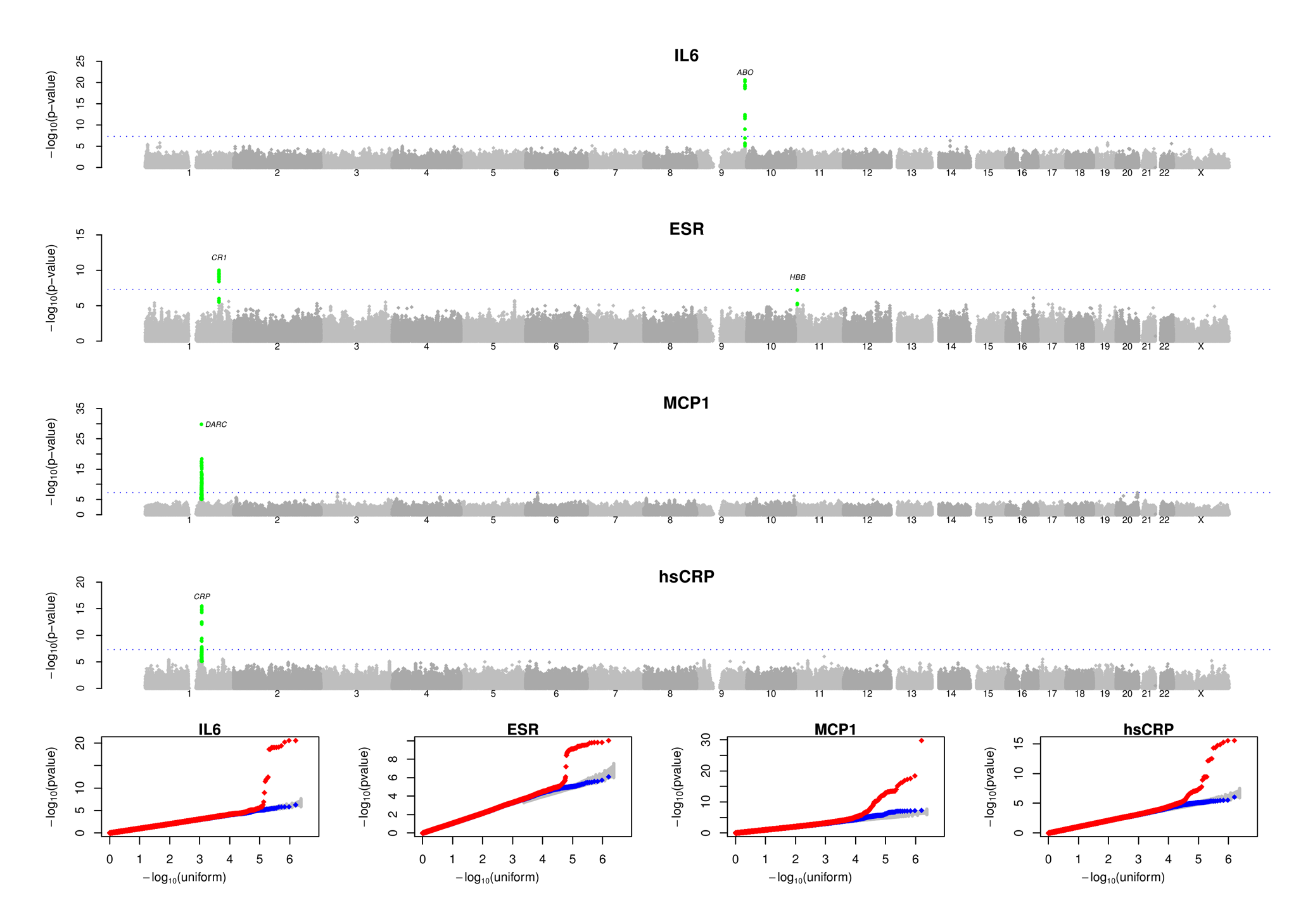

Supplement: Figure S1 — Manhattan plot and QQ plot of association findings in step 1 GWAS. The figure summarizes the genome-wide association scan results combined across the data-sets by inverse variance weighting. The blue dotted line marks the threshold for genome-wide significance (5×10^−08^) [64]. SNPs in loci exceeding this threshold are highlighted in green. The bottom panel represents the QQ plot, where the red line corresponds to all test statistics, and the blue line to results after excluding statistics at top markers (highlighted in green in the Manhattan Plot). The gray area corresponds to the 90% confidence region from a null distribution of P values (generated from 100 simulations). (PNG) [file pgen.1002480.s001.png]

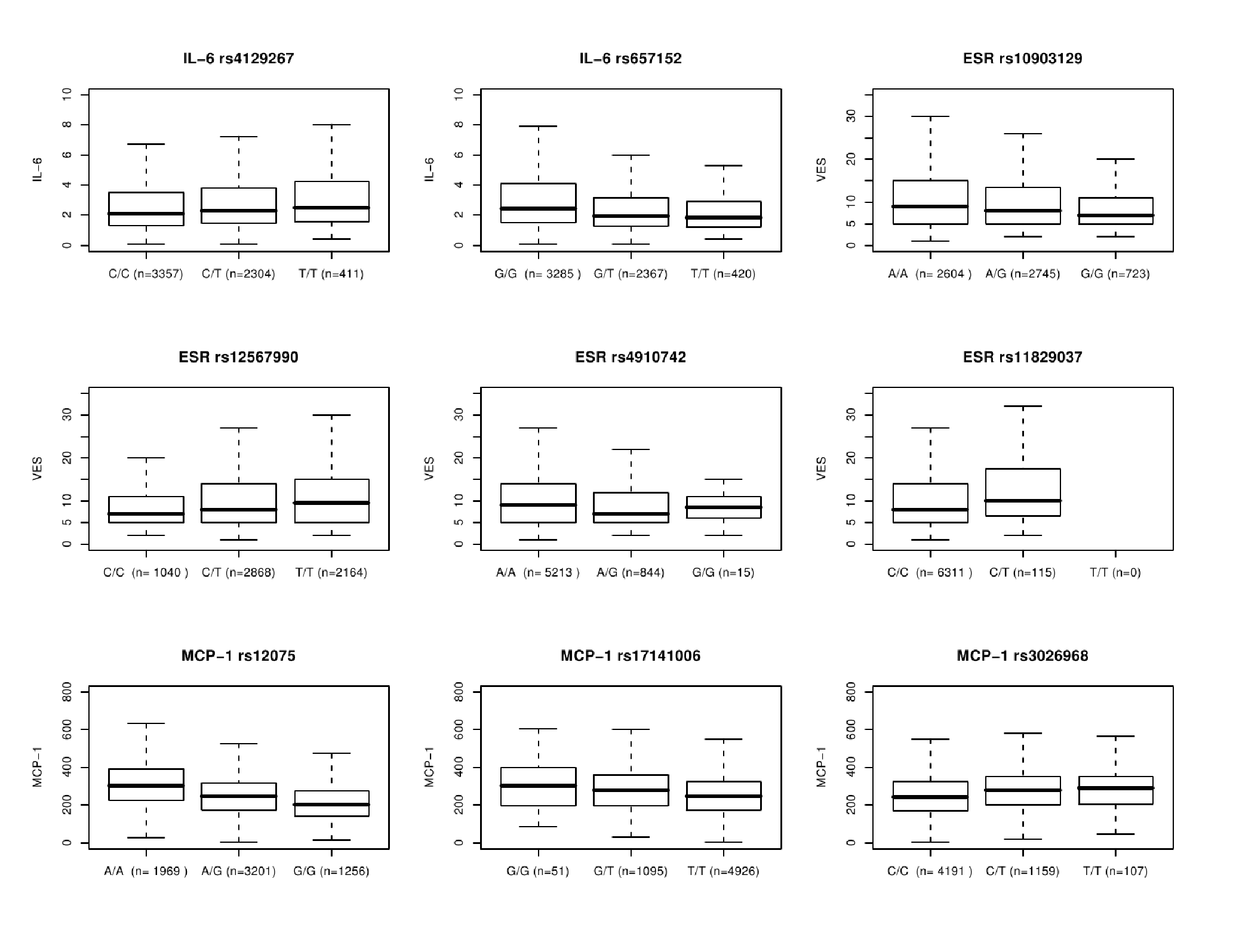

Supplement: Figure S2 — Boxplots for levels of IL-6, ESR, and MCP-1 for each genotype at the top associated SNPs. Standard boxplots are drawn with min, 0.25 quantile; median, 0.75 quantile; and 0.75 quantile+ 1.5*IQR for the levels of unadjusted biomarkers IL-6 (pg/ml), ESR (mm/h), and MCP-1 (pg/ml) in the original units. For each boxplot the name of the biomarker and the associated SNP are indicated, as well as the number of individuals per genotype. (PNG) [file pgen.1002480.s002.png]

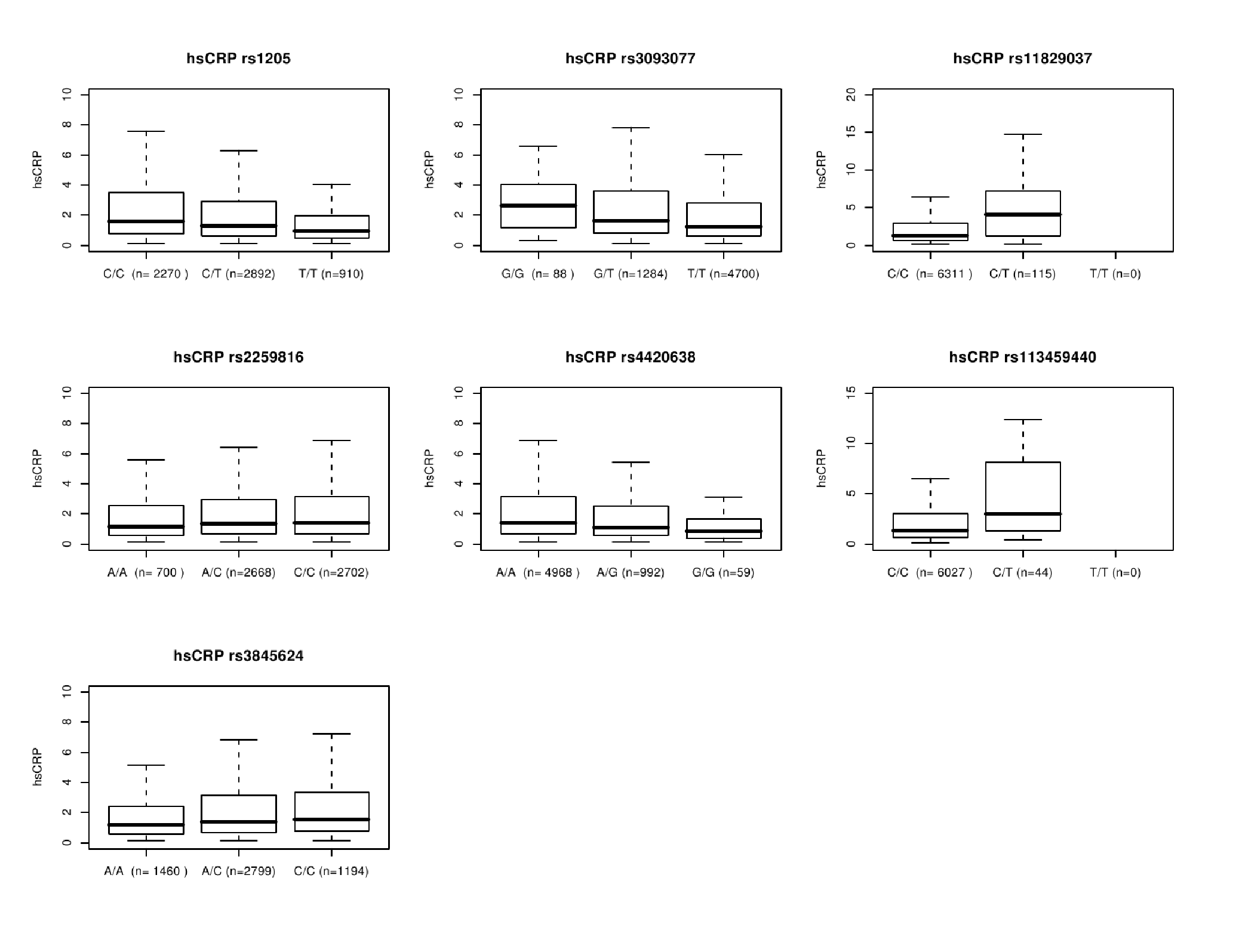

Supplement: Figure S3 — Boxplots for levels of hsCRP for each genotype at the top associated SNPs. Standard boxplots are drawn with min, 0.25 quantile; median, 0.75 quantile; and 0.75 quantile+ 1.5*IQR for the levels of unadjusted hs-CRP (mg/ml) biomarker in the original units. For each boxplot the name of the biomarker and the associated SNP are indicated, as well as the number of individuals per genotype. (PNG) [file pgen.1002480.s003.png]
