# Supplementary material for: A Genome-Wide Association Scan on the Levels of Markers of Inflammation in Sardinians Reveals Associations That Underpin Its Complex Regulation
Source: PLoS Genet. 2012 Jan 26;8(1):e1002480. doi: 10.1371/journal.pgen.1002480 (PMC3266885; doi:10.1371/journal.pgen.1002480)
Supplement: Table S1 — Association results for SNPs with p-value<10−5 for inflammatory biomarkers in the step 1 GWAS. The table summarizes the association results at SNPs with p-value<10−5 for each inflammatory biomarkers. The effect size is measured in standard deviations (e.g. an effect size of 1.0 implies each additional copy of the allele being evaluated increases trait values by 1.0 standard deviations) and it refers to allele 1. The r2 between each imputed genotype and the true underlying genotype is provided (RSQ) and serves as a quality-control metric. The percentage of the variance explained by each markers is also reported (H2), and the column “I/G” indicates whether the SNP has been imputed or genotyped. Physical positions are given according to build 36. SNPs above genome-wide threshold (p<5×10^−8^) are indicated in bold. EA, effect allele; OA, other allele. (DOC) [file pgen.1002480.s004.doc]

**Table S1. Association results for SNPs with p-value <10^-5^ for inflammatory biomarkers in the step 1 GWAS.**

| **Marker** | **Chr** | **Position** | **EA** | **OA** | **Freq EA** | **Effect** | **SE** | **H2** | **P** | **I/G** | **RSQR** |
| --- | --- | --- | --- | --- | --- | --- | --- | --- | --- | --- | --- |
| ***IL-6*** |  |  |  |  |  |  |  |  |  |  |  |
| **rs643434** | **9** | **135132176** | **G** | **A** | **0.742** | **0.245** | **0.026** | **2.813** | **2.69X10-21** | **I** | **0.999** |
| **rs644234** | **9** | **135132038** | **T** | **G** | **0.742** | **0.245** | **0.026** | **2.813** | **2.69X10-21** | **I** | **0.999** |
| **rs657152** | **9** | **135129086** | **C** | **A** | **0.743** | **0.243** | **0.026** | **2.76** | **6.04X10-21** | **G** | **-** |
| **rs529565** | **9** | **135139321** | **T** | **C** | **0.769** | **0.247** | **0.027** | **2.659** | **3.85X10-20** | **I** | **0.967** |
| **rs505922** | **9** | **135139050** | **T** | **C** | **0.769** | **0.245** | **0.027** | **2.615** | **7.71X10-20** | **I** | **0.975** |
| **rs674302** | **9** | **135136485** | **T** | **A** | **0.769** | **0.245** | **0.027** | **2.611** | **8.41X10-20** | **I** | **0.986** |
| **rs545971** | **9** | **135133193** | **C** | **T** | **0.769** | **0.245** | **0.027** | **2.607** | **8.99X10-20** | **I** | **0.999** |
| **rs612169** | **9** | **135133263** | **A** | **G** | **0.769** | **0.245** | **0.027** | **2.607** | **8.99X10-20** | **G** | **-** |
| **rs514659** | **9** | **135132024** | **A** | **C** | **0.769** | **0.245** | **0.027** | **2.607** | **9.04X10-20** | **I** | **0.999** |
| **rs687621** | **9** | **135126886** | **A** | **G** | **0.770** | **0.242** | **0.027** | **2.545** | **2.35X10-19** | **I** | **0.947** |
| **rs687289** | **9** | **135126927** | **G** | **A** | **0.770** | **0.242** | **0.027** | **2.545** | **2.36X10-19** | **I** | **0.953** |
| **rs579459** | **9** | **135143989** | **T** | **C** | **0.826** | **0.201** | **0.028** | **1.422** | **3.63X10-13** | **I** | **0.963** |
| **rs649129** | **9** | **135144125** | **C** | **T** | **0.826** | **0.217** | **0.030** | **1.654** | **7.81X10-13** | **I** | **0.933** |
| **rs495828** | **9** | **135144688** | **G** | **T** | **0.826** | **0.215** | **0.030** | **1.617** | **1.30X10-12** | **I** | **0.919** |
| **rs651007** | **9** | **135143696** | **C** | **T** | **0.827** | **0.211** | **0.030** | **1.551** | **3.35X10-12** | **G** | **-** |
| **rs630014** | **9** | **135139543** | **A** | **G** | **0.623** | **0.143** | **0.023** | **1.170** | **1.08X10-09** | **I** | **0.914** |
| rs3758348 | 9 | 135229220 | G | C | 0.859 | 0.157 | 0.030 | 0.734 | 1.22X10-07 | I | 0.713 |
| rs1008924 | 14 | 51152830 | A | G | 0.507 | 0.111 | 0.022 | 0.748 | 5.55X10-07 | G | - |
| ***ESR*** |  |  |  |  |  |  |  |  |  |  |  |
| **rs12034598** | **1** | **205824138** | **G** | **A** | **0.589** | **0.143** | **0.022** | **1.411** | **9.31X10-11** | **G** | **-** |
| **rs12567990** | **1** | **205748308** | **T** | **C** | **0.592** | **0.141** | **0.022** | **1.368** | **1.50X10-10** | **G** | **-** |
| **rs12567973** | **1** | **205748124** | **G** | **C** | **0.592** | **0.141** | **0.022** | **1.368** | **1.50X10-10** | **I** | **0.962** |
| **rs11117959** | **1** | **205751142** | **G** | **A** | **0.592** | **0.141** | **0.022** | **1.368** | **1.50X10-10** | **I** | **0.996** |
| **rs4844599** | **1** | **205745852** | **G** | **T** | **0.592** | **0.140** | **0.022** | **1.361** | **1.63X10-10** | **I** | **0.93** |
| **rs2274567** | **1** | **205820244** | **G** | **A** | **0.591** | **0.140** | **0.022** | **1.357** | **1.72X10-10** | **I** | **0.994** |
| **rs11117956** | **1** | **205750982** | **G** | **T** | **0.593** | **0.139** | **0.022** | **1.337** | **2.56X10-10** | **I** | **0.985** |
| **rs17046851** | **1** | **205814607** | **G** | **A** | **0.595** | **0.139** | **0.022** | **1.342** | **2.84X10-10** | **I** | **0.991** |
| **rs11118131** | **1** | **205827819** | **T** | **C** | **0.592** | **0.138** | **0.022** | **1.321** | **3.00X10-10** | **I** | **0.994** |
| **rs3738468** | **1** | **205828981** | **A** | **G** | **0.592** | **0.138** | **0.022** | **1.321** | **3.03X10-10** | **I** | **0.995** |
| **rs10779330** | **1** | **205829297** | **A** | **G** | **0.592** | **0.138** | **0.022** | **1.321** | **3.04X10-10** | **I** | **0.995** |
| **rs10863358** | **1** | **205757494** | **C** | **G** | **0.596** | **0.138** | **0.022** | **1.319** | **3.57X10-10** | **I** | **0.985** |
| **rs646817** | **1** | **205824559** | **G** | **A** | **0.595** | **0.138** | **0.022** | **1.309** | **3.74X10-10** | **I** | **0.995** |
| **rs11118135** | **1** | **205837051** | **G** | **A** | **0.592** | **0.137** | **0.022** | **1.303** | **4.03X10-10** | **I** | **0.996** |
| **rs12141045** | **1** | **205838777** | **C** | **T** | **0.592** | **0.137** | **0.022** | **1.302** | **4.10X10-10** | **G** | **-** |
| **rs11118136** | **1** | **205839031** | **G** | **A** | **0.592** | **0.137** | **0.022** | **1.302** | **4.12X10-10** | **I** | **0.993** |
| **rs7519119** | **1** | **205852775** | **G** | **A** | **0.751** | **0.154** | **0.025** | **1.277** | **5.87X10-10** | **I** | **0.884** |
| **rs7539922** | **1** | **205839962** | **A** | **G** | **0.592** | **0.136** | **0.022** | **1.277** | **6.05X10-10** | **I** | **0.984** |
| **rs12757487** | **1** | **205815811** | **A** | **G** | **0.595** | **0.137** | **0.022** | **1.291** | **6.19X10-10** | **I** | **0.995** |
| **rs11118167** | **1** | **205848777** | **C** | **T** | **0.601** | **0.136** | **0.022** | **1.267** | **6.83X10-10** | **I** | **0.993** |
| **rs10429953** | **1** | **205855201** | **G** | **A** | **0.591** | **0.136** | **0.022** | **1.274** | **7.07X10-10** | **G** | **-** |
| **rs12034383** | **1** | **205870218** | **G** | **A** | **0.752** | **0.154** | **0.025** | **1.260** | **7.41X10-10** | **I** | **0.959** |
| **rs12041437** | **1** | **205869887** | **C** | **A** | **0.752** | **0.154** | **0.025** | **1.259** | **7.47X10-10** | **I** | **0.977** |
| **rs11803956** | **1** | **205869644** | **T** | **C** | **0.752** | **0.154** | **0.025** | **1.259** | **7.49X10-10** | **G** | **-** |
| **rs2274566** | **1** | **205819968** | **C** | **T** | **0.759** | **0.154** | **0.025** | **1.250** | **7.66X10-10** | **G** | **-** |
| **rs11118166** | **1** | **205848618** | **G** | **A** | **0.601** | **0.135** | **0.022** | **1.262** | **7.75X10-10** | **G** | **-** |
| **rs3886100** | **1** | **205805750** | **A** | **G** | **0.772** | **0.159** | **0.026** | **1.282** | **7.93X10-10** | **I** | **0.899** |
| **rs650877** | **1** | **205815416** | **G** | **A** | **0.596** | **0.136** | **0.022** | **1.280** | **8.98X10-10** | **G** | **-** |
| **rs6691117** | **1** | **205849554** | **G** | **A** | **0.602** | **0.135** | **0.022** | **1.246** | **1.01X10-09** | **I** | **0.988** |
| **rs7542544** | **1** | **205852846** | **C** | **A** | **0.753** | **0.152** | **0.025** | **1.233** | **1.07X10-09** | **I** | **0.973** |
| **rs677066** | **1** | **205840614** | **C** | **T** | **0.602** | **0.134** | **0.022** | **1.240** | **1.08X10-09** | **G** | **-** |
| **rs11118157** | **1** | **205844942** | **A** | **C** | **0.602** | **0.134** | **0.022** | **1.240** | **1.09X10-09** | **I** | **0.999** |
| **rs12032275** | **1** | **205850130** | **T** | **C** | **0.594** | **0.134** | **0.022** | **1.235** | **1.42X10-09** | **I** | **0.988** |
| **rs11803366** | **1** | **205873612** | **T** | **C** | **0.753** | **0.151** | **0.025** | **1.211** | **1.47X10-09** | **I** | **0.923** |
| **rs12734030** | **1** | **205860587** | **T** | **C** | **0.587** | **0.132** | **0.022** | **1.213** | **1.84X10-09** | **I** | **0.929** |
| **rs599948** | **1** | **205818862** | **C** | **T** | **0.598** | **0.132** | **0.022** | **1.198** | **2.22X10-09** | **G** | **-** |
| **rs614709** | **1** | **205819898** | **C** | **T** | **0.598** | **0.130** | **0.022** | **1.167** | **3.69X10-09** | **I** | **0.998** |
| **rs601356** | **1** | **205819173** | **G** | **T** | **0.598** | **0.130** | **0.022** | **1.167** | **3.71X10-09** | **I** | **0.999** |
| **rs4910742** | **11** | **5263085** | **A** | **G** | **0.934** | **0.229** | **0.042** | **0.929** | **6.34X10-08** | **G** | **-** |
| **rs11861089** | **16** | **76577188** | **T** | **C** | **0.694** | **-0.116** | **0.023** | **0.819** | **7.99X10-07** | **I** | **0.985** |
| ***MCP-1*** |  |  |  |  |  |  |  |  |  |  |  |
| **rs12075** | **1** | **157441978** | **G** | **A** | **0.510** | **-0.303** | **0.026** | **4.861** | **1.68X10-30** | **I** | **0.7099** |
| **rs2494261** | **1** | **157517918** | **A** | **G** | **0.738** | **0.252** | **0.028** | **2.611** | **4.04X10-19** | **I** | **0.927** |
| **rs2427832** | **1** | **157499699** | **G** | **A** | **0.719** | **0.238** | **0.027** | **2.434** | **2.81X10-18** | **I** | **0.949** |
| **rs2494258** | **1** | **157501203** | **C** | **T** | **0.717** | **0.237** | **0.027** | **2.423** | **5.74X10-18** | **I** | **0.886** |
| **rs863017** | **1** | **157481168** | **T** | **A** | **0.721** | **0.234** | **0.027** | **2.338** | **1.20X10-17** | **G** | **-** |
| **rs2511212** | **1** | **157511517** | **T** | **C** | **0.729** | **0.232** | **0.028** | **2.262** | **4.71X10-17** | **I** | **0.937** |
| **rs863011** | **1** | **157460796** | **T** | **C** | **0.719** | **0.226** | **0.027** | **2.196** | **8.26X10-17** | **G** | **-** |
| **rs2494260** | **1** | **157512809** | **C** | **T** | **0.733** | **0.23** | **0.028** | **2.195** | **2.59X10-16** | **I** | **0.878** |
| **rs1446954** | **1** | **157862753** | **G** | **C** | **0.819** | **0.249** | **0.031** | **1.946** | **7.40X10-16** | **G** | **-** |
| **rs3027012** | **1** | **157440747** | **C** | **T** | **0.886** | **-0.302** | **0.039** | **1.964** | **8.92X10-15** | **G** | **-** |
| **rs11265186** | **1** | **157642527** | **C** | **T** | **0.886** | **0.292** | **0.039** | **1.834** | **3.31X10-14** | **G** | **-** |
| **rs10908717** | **1** | **157642976** | **C** | **A** | **0.886** | **0.292** | **0.039** | **1.834** | **3.31X10-14** | **I** | **0.998** |
| **rs10908716** | **1** | **157642967** | **A** | **G** | **0.886** | **0.292** | **0.039** | **1.834** | **3.31X10-14** | **I** | **0.999** |
| **rs11265187** | **1** | **157645218** | **C** | **T** | **0.886** | **0.292** | **0.039** | **1.825** | **3.87X10-14** | **G** | **-** |
| **rs4492615** | **1** | **157658487** | **T** | **C** | **0.886** | **0.292** | **0.039** | **1.826** | **3.95X10-14** | **G** | **-** |
| **rs4656237** | **1** | **157680448** | **T** | **C** | **0.886** | **0.290** | **0.038** | **1.806** | **4.08X10-14** | **I** | **0.984** |
| **rs12410729** | **1** | **157680658** | **T** | **C** | **0.884** | **0.287** | **0.038** | **1.794** | **5.07X10-14** | **I** | **0.926** |
| **rs11265193** | **1** | **157668280** | **G** | **T** | **0.886** | **0.289** | **0.038** | **1.799** | **5.39X10-14** | **I** | **0.994** |
| **rs4128725** | **1** | **157672583** | **T** | **C** | **0.886** | **0.289** | **0.038** | **1.798** | **5.46X10-14** | **G** | **-** |
| **rs12118628** | **1** | **157676508** | **G** | **A** | **0.886** | **0.289** | **0.038** | **1.796** | **5.64X10-14** | **I** | **0.989** |
| **rs12087465** | **1** | **157891923** | **C** | **A** | **0.790** | **0.219** | **0.029** | **1.685** | **8.05X10-14** | **I** | **0.983** |
| **rs12142553** | **1** | **157608296** | **T** | **C** | **0.884** | **0.283** | **0.038** | **1.744** | **1.12X10-13** | **I** | **0.978** |
| **rs2592881** | **1** | **157871724** | **T** | **C** | **0.790** | **0.222** | **0.030** | **1.74** | **1.19X10-13** | **I** | **0.958** |
| **rs4399156** | **1** | **157604696** | **G** | **A** | **0.884** | **0.283** | **0.038** | **1.74** | **1.21X10-13** | **I** | **0.937** |
| **rs12060842** | **1** | **157865371** | **G** | **A** | **0.800** | **0.218** | **0.030** | **1.612** | **5.09X10-13** | **I** | **0.970** |
| **rs11265206** | **1** | **157715707** | **T** | **C** | **0.886** | **0.277** | **0.038** | **1.651** | **5.53X10-13** | **G** | **-** |
| **rs6660102** | **1** | **157477688** | **G** | **T** | **0.861** | **-0.258** | **0.036** | **1.69** | **5.72X10-13** | **I** | **0.909** |
| **rs12072644** | **1** | **157481911** | **C** | **T** | **0.861** | **-0.258** | **0.036** | **1.693** | **6.70X10-13** | **I** | **0.917** |
| **rs11265218** | **1** | **157753264** | **T** | **C** | **0.884** | **0.260** | **0.036** | **1.463** | **7.67X10-13** | **I** | **0.868** |
| **rs1446957** | **1** | **157863390** | **C** | **G** | **0.801** | **0.223** | **0.031** | **1.688** | **7.99X10-13** | **I** | **0.967** |
| **rs10908724** | **1** | **157716792** | **G** | **T** | **0.886** | **0.274** | **0.038** | **1.613** | **9.79X10-13** | **I** | **0.975** |
| **rs12143656** | **1** | **157745772** | **A** | **G** | **0.885** | **0.275** | **0.039** | **1.638** | **1.17X10-12** | **I** | **0.864** |
| **rs12142583** | **1** | **157744571** | **A** | **G** | **0.886** | **0.272** | **0.039** | **1.589** | **1.49X10-12** | **I** | **0.866** |
| **rs2794525** | **1** | **157869139** | **G** | **A** | **0.750** | **0.200** | **0.028** | **1.587** | **1.73X10-12** | **I** | **0.975** |
| **rs12405354** | **1** | **157504276** | **A** | **G** | **0.860** | **-0.254** | **0.036** | **1.646** | **1.97X10-12** | **G** | **-** |
| **rs2427825** | **1** | **157532690** | **C** | **T** | **0.677** | **0.186** | **0.027** | **1.603** | **3.19X10-12** | **I** | **0.972** |
| **rs2808652** | **1** | **157872880** | **T** | **C** | **0.749** | **0.197** | **0.028** | **1.541** | **3.60X10-12** | **G** | **-** |
| **rs10908733** | **1** | **157770678** | **T** | **A** | **0.871** | **0.245** | **0.036** | **1.432** | **9.86X10-12** | **I** | **0.938** |
| **rs2427824** | **1** | **157531686** | **C** | **T** | **0.678** | **0.181** | **0.027** | **1.519** | **1.33X10-11** | **I** | **0.959** |
| **rs2494263** | **1** | **157522418** | **A** | **G** | **0.680** | **0.181** | **0.027** | **1.506** | **1.87X10-11** | **I** | **0.904** |
| **rs1446958** | **1** | **157866431** | **A** | **G** | **0.754** | **0.191** | **0.029** | **1.430** | **2.48X10-11** | **I** | **0.947** |
| **rs1867939** | **1** | **157872518** | **A** | **C** | **0.754** | **0.190** | **0.029** | **1.418** | **2.81X10-11** | **G** | **-** |
| **rs984553** | **1** | **157866038** | **A** | **G** | **0.753** | **0.190** | **0.029** | **1.421** | **2.98X10-11** | **I** | **0.953** |
| **rs17666424** | **1** | **157454154** | **G** | **A** | **0.913** | **-0.296** | **0.045** | **1.468** | **3.22X10-11** | **G** | **-** |
| **rs2794514** | **1** | **157864927** | **A** | **G** | **0.732** | **0.186** | **0.028** | **1.435** | **3.73X10-11** | **I** | **0.906** |
| **rs2494250** | **1** | **157544875** | **C** | **G** | **0.677** | **0.174** | **0.027** | **1.409** | **5.23X10-11** | **I** | **0.961** |
| **rs2808653** | **1** | **157873044** | **C** | **T** | **0.754** | **0.187** | **0.029** | **1.376** | **5.64X10-11** | **I** | **0.993** |
| **rs2808651** | **1** | **157872850** | **T** | **C** | **0.754** | **0.187** | **0.029** | **1.376** | **5.65X10-11** | **G** | **-** |
| **rs985472** | **1** | **157875569** | **T** | **A** | **0.754** | **0.187** | **0.029** | **1.374** | **5.75X10-11** | **I** | **0.974** |
| **rs983789** | **1** | **157862306** | **G** | **T** | **0.792** | **0.203** | **0.031** | **1.437** | **6.24X10-11** | **I** | **0.933** |
| **rs2427828** | **1** | **157539665** | **A** | **G** | **0.679** | **0.173** | **0.027** | **1.391** | **7.07X10-11** | **G** | **-** |
| **rs2494265** | **1** | **157541898** | **T** | **A** | **0.679** | **0.173** | **0.027** | **1.389** | **7.28X10-11** | **I** | **0.982** |
| **rs41497649** | **1** | **157891381** | **G** | **A** | **0.818** | **0.203** | **0.031** | **1.311** | **7.83X10-11** | **G** | **-** |
| **rs11265230** | **1** | **157781919** | **A** | **G** | **0.869** | **0.235** | **0.036** | **1.328** | **8.98X10-11** | **G** | **-** |
| **rs1446955** | **1** | **157862780** | **G** | **A** | **0.798** | **0.194** | **0.03** | **1.289** | **9.28X10-11** | **I** | **0.965** |
| **rs12047230** | **1** | **157745629** | **G** | **A** | **0.619** | **0.166** | **0.026** | **1.385** | **1.03X10-10** | **I** | **0.888** |
| **rs1584250** | **1** | **157774298** | **C** | **T** | **0.870** | **0.230** | **0.036** | **1.278** | **2.20X10-10** | **I** | **0.975** |
| **rs3026968** | **1** | **157414076** | **C** | **T** | **0.869** | **-0.235** | **0.037** | **1.331** | **2.63X10-10** | **I** | **0.864** |
| **rs1584252** | **1** | **157790252** | **T** | **A** | **0.594** | **0.158** | **0.025** | **1.283** | **4.10X10-10** | **G** | **-** |
| **rs1474747** | **1** | **157415137** | **T** | **C** | **0.605** | **0.160** | **0.026** | **1.300** | **5.74X10-10** | **I** | **0.883** |
| **rs16841987** | **1** | **157531890** | **G** | **A** | **0.904** | **-0.230** | **0.038** | **0.982** | **1.20X10-09** | **I** | **0.932** |
| **rs11265142** | **1** | **157366532** | **A** | **G** | **0.891** | **0.243** | **0.040** | **1.223** | **1.28X10-09** | **I** | **0.968** |
| **rs11265180** | **1** | **157610272** | **G** | **C** | **0.861** | **0.208** | **0.034** | **1.096** | **1.53X10-09** | **I** | **0.836** |
| **rs965358** | **1** | **157406973** | **A** | **T** | **0.617** | **0.155** | **0.026** | **1.201** | **1.59X10-09** | **G** | **-** |
| **rs11265177** | **1** | **157592458** | **A** | **G** | **0.738** | **0.171** | **0.029** | **1.195** | **2.27X10-09** | **G** | **-** |
| **rs9782926** | **1** | **157590016** | **T** | **A** | **0.738** | **0.168** | **0.028** | **1.159** | **2.55X10-09** | **I** | **0.994** |
| **rs2281300** | **1** | **157422909** | **C** | **T** | **0.873** | **-0.226** | **0.038** | **1.207** | **3.20X10-09** | **I** | **0.628** |
| **rs3027031** | **1** | **157444897** | **C** | **G** | **0.925** | **-0.284** | **0.048** | **1.187** | **3.97X10-09** | **I** | **0.909** |
| **rs2518569** | **1** | **157362199** | **G** | **T** | **0.892** | **0.237** | **0.04** | **1.149** | **4.62X10-09** | **I** | **0.864** |
| **rs7522607** | **1** | **157552615** | **T** | **C** | **0.904** | **-0.225** | **0.039** | **0.936** | **5.78X10-09** | **I** | **0.953** |
| **rs1446956** | **1** | **157863345** | **T** | **C** | **0.733** | **0.163** | **0.028** | **1.098** | **5.87X10-09** | **I** | **0.941** |
| **rs7549785** | **1** | **157544492** | **G** | **A** | **0.904** | **-0.225** | **0.039** | **0.935** | **5.94X10-09** | **G** | **-** |
| **rs3845625** | **1** | **157532500** | **C** | **T** | **0.904** | **-0.249** | **0.043** | **1.148** | **6.31X10-09** | **I** | **0.935** |
| **rs2427834** | **1** | **157513147** | **G** | **A** | **0.723** | **0.222** | **0.038** | **2.092** | **6.78X10-09** | **G** | **-** |
| **rs1446971** | **1** | **157851788** | **G** | **A** | **0.794** | **0.173** | **0.030** | **1.040** | **1.04X10-08** | **I** | **0.913** |
| **rs16841832** | **1** | **157420936** | **G** | **A** | **0.873** | **-0.212** | **0.037** | **1.057** | **1.11X10-08** | **G** | **-** |
| **rs11265174** | **1** | **157586983** | **T** | **A** | **0.737** | **0.160** | **0.028** | **1.055** | **1.15X10-08** | **I** | **0.974** |
| **rs4269772** | **1** | **157557285** | **T** | **C** | **0.734** | **0.160** | **0.028** | **1.056** | **1.30X10-08** | **I** | **0.947** |
| **rs10489849** | **1** | **157463389** | **C** | **T** | **0.834** | **-0.185** | **0.033** | **1.001** | **2.48X10-08** | **G** | **-** |
| **rs4446959** | **1** | **157624308** | **T** | **C** | **0.805** | **0.174** | **0.031** | **1.013** | **2.69X10-08** | **G** | **-** |
| **rs11265194** | **1** | **157670313** | **T** | **G** | **0.811** | **0.170** | **0.031** | **0.941** | **3.87X10-08** | **I** | **0.933** |
| **rs3806185** | **1** | **158015510** | **C** | **T** | **0.788** | **0.167** | **0.030** | **0.986** | **3.99X10-08** | **G** | **-** |
| **rs3026946** | **1** | **157401906** | **C** | **T** | **0.862** | **-0.20** | **0.036** | **1.009** | **4.19X10-08** | **I** | **0.701** |
| rs6513566 | 20 | 59233504 | C | A | 0.633 | -0.139 | 0.026 | 0.951 | 5.29X10-08 | I | 0.975 |
| rs9405112 | 6 | 32553578 | G | A | 0.758 | 0.157 | 0.029 | 0.958 | 6.43X10-08 | I | 0.806 |
| rs6427482 | 1 | 157765882 | A | G | 0.747 | 0.154 | 0.029 | 0.957 | 7.37X10-08 | I | 0.928 |
| rs9268858 | 6 | 32537736 | T | C | 0.739 | 0.156 | 0.029 | 1.001 | 7.68X10-08 | G | - |
| rs3026943 | 1 | 157398550 | A | C | 0.556 | -0.185 | 0.035 | 1.802 | 8.15X10-08 | G | - |
| rs2395185 | 6 | 32541145 | G | T | 0.739 | 0.156 | 0.029 | 0.998 | 8.38X10-08 | I | 0.918 |
| rs3918357 | 3 | 46368974 | C | A | 0.904 | -0.221 | 0.041 | 0.904 | 8.49X10-08 | G | - |
| rs9268853 | 6 | 32537621 | T | C | 0.739 | 0.155 | 0.029 | 0.984 | 9.16X10-08 | G | - |
| rs9405040 | 6 | 32547371 | A | C | 0.739 | 0.156 | 0.029 | 0.991 | 9.19X10-08 | I | 0.853 |
| rs9286790 | 6 | 32547806 | G | A | 0.739 | 0.156 | 0.029 | 0.990 | 9.25X10-08 | I | 0.843 |
| rs9268969 | 6 | 32542327 | C | T | 0.739 | 0.155 | 0.029 | 0.990 | 9.38X10-08 | I | 0.879 |
| rs9268923 | 6 | 32540813 | C | T | 0.739 | 0.155 | 0.029 | 0.986 | 9.85X10-08 | I | 0.907 |
| rs4517343 | 1 | 157605630 | A | G | 0.806 | 0.166 | 0.031 | 0.915 | 1.25X10-07 | I | 0.921 |
| rs4537549 | 1 | 157605660 | C | G | 0.806 | 0.166 | 0.031 | 0.915 | 1.25X10-07 | I | 0.926 |
| rs6677678 | 1 | 157606144 | C | T | 0.806 | 0.166 | 0.031 | 0.915 | 1.25X10-07 | I | 0.936 |
| rs12048482 | 1 | 157676481 | G | A | 0.754 | 0.151 | 0.029 | 0.901 | 1.44X10-07 | I | 0.966 |
| rs1891490 | 20 | 59236556 | C | T | 0.637 | -0.134 | 0.026 | 0.883 | 1.70X10-07 | G | - |
| rs4400605 | 1 | 157607040 | T | C | 0.806 | 0.162 | 0.031 | 0.871 | 1.78X10-07 | I | 0.906 |
| rs11265196 | 1 | 157684836 | A | G | 0.755 | 0.150 | 0.029 | 0.889 | 1.80X10-07 | G | - |
| rs10908713 | 1 | 157606183 | T | C | 0.806 | 0.162 | 0.031 | 0.869 | 1.82X10-07 | I | 0.941 |
| rs6701545 | 1 | 157618591 | T | C | 0.807 | 0.161 | 0.031 | 0.863 | 1.89X10-07 | I | 0.940 |
| rs3122633 | 1 | 157644082 | T | C | 0.809 | 0.161 | 0.031 | 0.848 | 1.94X10-07 | I | 0.937 |
| rs4130236 | 1 | 157608475 | C | T | 0.806 | 0.164 | 0.032 | 0.892 | 1.95X10-07 | I | 0.954 |
| rs6129076 | 20 | 59221046 | T | C | 0.627 | -0.132 | 0.025 | 0.867 | 2.01X10-07 | I | 0.994 |
| rs2426990 | 20 | 59221398 | A | G | 0.627 | -0.132 | 0.025 | 0.867 | 2.02X10-07 | G | - |
| rs4661129 | 1 | 155589420 | C | T | 0.757 | -0.151 | 0.029 | 0.887 | 2.03X10-07 | G | - |
| rs1857198 | 20 | 59219645 | T | C | 0.628 | -0.132 | 0.025 | 0.867 | 2.06X10-07 | I | 0.984 |
| rs11265191 | 1 | 157666983 | C | T | 0.753 | 0.149 | 0.029 | 0.876 | 2.09X10-07 | I | 0.943 |
| rs6129077 | 20 | 59224737 | G | C | 0.626 | -0.131 | 0.025 | 0.858 | 2.36X10-07 | I | 0.996 |
| rs6028074 | 20 | 59225366 | G | C | 0.626 | -0.131 | 0.025 | 0.858 | 2.37X10-07 | I | 0.995 |
| rs11697871 | 20 | 59227434 | C | T | 0.626 | -0.131 | 0.025 | 0.853 | 2.55X10-07 | I | 0.994 |
| rs6687840 | 1 | 157609063 | C | T | 0.807 | 0.162 | 0.032 | 0.873 | 2.69X10-07 | I | 0.945 |
| rs2794506 | 1 | 157852184 | A | G | 0.715 | 0.142 | 0.028 | 0.870 | 3.02X10-07 | G | - |
| rs16842599 | 1 | 157964099 | T | C | 0.881 | 0.194 | 0.038 | 0.836 | 3.02X10-07 | G | - |
| rs6065074 | 20 | 59229612 | T | G | 0.626 | -0.130 | 0.025 | 0.840 | 3.11X10-07 | G | - |
| rs11811256 | 1 | 157744854 | C | T | 0.749 | 0.146 | 0.029 | 0.853 | 3.30X10-07 | I | 0.921 |
| rs6702853 | 1 | 157609034 | T | A | 0.806 | 0.158 | 0.031 | 0.825 | 3.54X10-07 | I | 0.949 |
| rs3093075 | 1 | 157946537 | G | T | 0.878 | 0.191 | 0.038 | 0.833 | 3.62X10-07 | I | 0.998 |
| rs3093077 | 1 | 157946260 | A | C | 0.878 | 0.191 | 0.038 | 0.832 | 3.65X10-07 | G | - |
| rs6015934 | 20 | 59233455 | A | G | 0.625 | -0.129 | 0.025 | 0.833 | 3.70X10-07 | I | 0.983 |
| rs16842568 | 1 | 157942844 | A | G | 0.878 | 0.190 | 0.038 | 0.826 | 4.04X10-07 | I | 0.991 |
| rs16842559 | 1 | 157942795 | T | C | 0.878 | 0.190 | 0.038 | 0.825 | 4.05X10-07 | I | 0.989 |
| rs11265190 | 1 | 157658025 | A | G | 0.754 | 0.146 | 0.029 | 0.839 | 4.13X10-07 | I | 0.939 |
| rs12034969 | 1 | 157651568 | C | T | 0.754 | 0.146 | 0.029 | 0.837 | 4.32X10-07 | I | 0.939 |
| rs16842502 | 1 | 157920487 | C | A | 0.879 | 0.187 | 0.037 | 0.784 | 4.42X10-07 | I | 0.902 |
| rs6692378 | 1 | 157605865 | T | C | 0.751 | 0.145 | 0.029 | 0.829 | 4.52X10-07 | I | 0.946 |
| rs983788 | 1 | 157862041 | A | G | 0.715 | 0.140 | 0.028 | 0.843 | 4.76X10-07 | I | 0.977 |
| rs12771415 | 10 | 131885351 | T | A | 0.921 | 0.220 | 0.044 | 0.745 | 6.02X10-07 | G | - |
| rs6081741 | 20 | 19692341 | A | G | 0.652 | -0.134 | 0.027 | 0.866 | 6.20X10-07 | I | 0.812 |
| rs12145616 | 1 | 157676103 | T | G | 0.881 | 0.227 | 0.046 | 1.146 | 6.58X10-07 | G | - |
| rs12068753 | 1 | 157959161 | T | A | 0.877 | 0.184 | 0.037 | 0.779 | 7.09X10-07 | G | - |
| rs9268979 | 6 | 32543022 | T | C | 0.675 | 0.135 | 0.027 | 0.854 | 7.52X10-07 | I | 0.839 |
| rs1500005 | 3 | 46077624 | G | A | 0.659 | 0.128 | 0.026 | 0.778 | 8.28X10-07 | I | 0.985 |
| rs2866004 | 20 | 59258437 | A | G | 0.686 | -0.131 | 0.027 | 0.780 | 8.32X10-07 | I | 0.941 |
| rs1572970 | 1 | 157940209 | A | G | 0.768 | 0.146 | 0.030 | 0.805 | 8.45X10-07 | I | 0.863 |
| ***hsCRP*** |  |  |  |  |  |  |  |  |  |  |  |
| **rs1341665** | **1** | **157958183** | **G** | **A** | **0.583** | **0.195** | **0.024** | **2.197** | **2.82X10-16** | **I** | **0.963** |
| **rs2027471** | **1** | **157956012** | **T** | **A** | **0.583** | **0.195** | **0.024** | **2.194** | **2.97X10-16** | **I** | **0.954** |
| **rs1205** | **1** | **157948857** | **C** | **T** | **0.585** | **0.193** | **0.024** | **2.159** | **5.42X10-16** | **I** | **0.971** |
| **rs7553007** | **1** | **157965173** | **G** | **A** | **0.603** | **0.191** | **0.024** | **2.078** | **1.41X10-15** | **G** | **-** |
| **rs2794520** | **1** | **157945440** | **C** | **T** | **0.595** | **0.190** | **0.024** | **2.065** | **1.82X10-15** | **G** | **-** |
| **rs2808628** | **1** | **157942635** | **G** | **A** | **0.595** | **0.187** | **0.024** | **2.003** | **4.40X10-15** | **I** | **0.968** |
| **rs2808629** | **1** | **157943420** | **G** | **A** | **0.594** | **0.186** | **0.024** | **1.993** | **5.05X10-15** | **G** | **-** |
| **rs2808624** | **1** | **157932545** | **C** | **G** | **0.541** | **0.171** | **0.023** | **1.730** | **3.17X10-13** | **I** | **0.955** |
| **rs11265257** | **1** | **157935608** | **C** | **T** | **0.542** | **0.171** | **0.024** | **1.726** | **3.80X10-13** | **I** | **0.940** |
| **rs1470515** | **1** | **157920223** | **C** | **T** | **0.547** | **0.171** | **0.024** | **1.713** | **6.31X10-13** | **I** | **0.899** |
| **rs2592887** | **1** | **157919563** | **C** | **T** | **0.540** | **0.172** | **0.024** | **1.748** | **7.16X10-13** | **I** | **0.885** |
| **rs3093077** | **1** | **157946260** | **A** | **C** | **0.878** | **-0.222** | **0.035** | **1.259** | **3.59X10-10** | **G** | **-** |
| **rs3093075** | **1** | **157946537** | **G** | **T** | **0.878** | **-0.222** | **0.035** | **1.259** | **3.59X10-10** | **I** | **0.998** |
| **rs16842568** | **1** | **157942844** | **A** | **G** | **0.878** | **-0.222** | **0.035** | **1.257** | **3.68X10-10** | **I** | **0.991** |
| **rs16842559** | **1** | **157942795** | **T** | **C** | **0.878** | **-0.222** | **0.035** | **1.257** | **3.68X10-10** | **I** | **0.989** |
| **rs3845624** | **1** | **157484890** | **A** | **C** | **0.523** | **-0.146** | **0.024** | **1.272** | **9.54X10-10** | **I** | **0.954** |
| **rs12068753** | **1** | **157959161** | **T** | **A** | **0.877** | **-0.213** | **0.035** | **1.162** | **1.20X10-09** | **G** | **-** |
| **rs16842502** | **1** | **157920487** | **C** | **A** | **0.879** | **-0.196** | **0.035** | **0.971** | **1.64X10-08** | **I** | **0.902** |
| **rs895581** | **1** | **157904611** | **A** | **G** | **0.747** | **-0.149** | **0.027** | **1.001** | **2.36X10-08** | **I** | **0.997** |
| **rs16842599** | **1** | **157964099** | **T** | **C** | **0.881** | **-0.198** | **0.036** | **0.977** | **2.85X10-08** | **G** | **-** |
| **rs895582** | **1** | **157904543** | **G** | **A** | **0.746** | **-0.145** | **0.026** | **0.951** | **3.23X10-08** | **I** | **0.996** |
| **rs2794498** | **1** | **157902740** | **G** | **T** | **0.746** | **-0.147** | **0.027** | **0.977** | **3.38X10-08** | **I** | **0.996** |
| **rs2808660** | **1** | **157882783** | **G** | **A** | **0.760** | **-0.147** | **0.027** | **0.940** | **4.00X10-08** | **I** | **0.964** |
| rs2592900 | 1 | 157883253 | A | G | 0.754 | -0.145 | 0.027 | 0.933 | 5.44X10-08 | I | 0.971 |
| rs1446974 | 1 | 157896008 | T | C | 0.755 | -0.143 | 0.026 | 0.898 | 6.37X10-08 | I | 0.992 |
| rs1971863 | 1 | 157905555 | T | C | 0.747 | -0.142 | 0.026 | 0.908 | 6.39X10-08 | G | - |
| rs1446977 | 1 | 157885980 | A | T | 0.759 | -0.145 | 0.027 | 0.910 | 6.92X10-08 | G | - |
| rs2592901 | 1 | 157883787 | C | T | 0.759 | -0.144 | 0.027 | 0.905 | 7.57X10-08 | I | 0.984 |
| rs16842484 | 1 | 157913548 | T | C | 0.734 | -0.140 | 0.026 | 0.907 | 7.71X10-08 | G | - |
| rs12118201 | 1 | 157663565 | C | T | 0.745 | 0.146 | 0.027 | 0.957 | 8.76X10-08 | I | 0.857 |
| rs12744244 | 1 | 157914280 | C | A | 0.768 | -0.145 | 0.027 | 0.889 | 8.78X10-08 | G | - |
| rs12081252 | 1 | 157973137 | T | C | 0.889 | -0.198 | 0.037 | 0.926 | 9.84X10-08 | I | 0.988 |
| rs12081264 | 1 | 157973184 | T | C | 0.889 | -0.198 | 0.037 | 0.926 | 9.86X10-08 | I | 0.982 |
| rs12755606 | 1 | 157936960 | C | G | 0.685 | -0.136 | 0.026 | 0.955 | 9.99X10-08 | I | 0.853 |
| rs1446975 | 1 | 157895966 | T | C | 0.537 | -0.124 | 0.023 | 0.913 | 1.03X10-07 | I | 0.995 |
| rs2794500 | 1 | 157901645 | C | T | 0.537 | -0.125 | 0.023 | 0.917 | 1.05X10-07 | G | - |
| rs12741825 | 1 | 157936769 | C | T | 0.696 | -0.135 | 0.026 | 0.917 | 1.34X10-07 | I | 0.912 |
| rs1891186 | 1 | 157895645 | A | G | 0.538 | -0.123 | 0.023 | 0.888 | 1.42X10-07 | I | 0.989 |
| rs11265260 | 1 | 157966663 | A | G | 0.889 | -0.194 | 0.037 | 0.883 | 1.50X10-07 | G | - |
| rs1446976 | 1 | 157895818 | A | G | 0.538 | -0.122 | 0.023 | 0.884 | 1.52X10-07 | I | 0.991 |
| rs863013 | 1 | 157466644 | G | T | 0.752 | 0.142 | 0.027 | 0.901 | 1.54X10-07 | I | 0.921 |
| rs7540542 | 1 | 157696951 | G | T | 0.726 | 0.136 | 0.026 | 0.882 | 1.91X10-07 | I | 0.955 |
| rs12749227 | 1 | 157925733 | C | T | 0.696 | -0.131 | 0.025 | 0.866 | 1.96X10-07 | I | 0.979 |
| rs12739022 | 1 | 157926055 | T | C | 0.696 | -0.131 | 0.025 | 0.866 | 1.97X10-07 | G | - |
| rs12754915 | 1 | 157927493 | T | C | 0.695 | -0.131 | 0.025 | 0.858 | 2.24X10-07 | I | 0.965 |
| rs12083620 | 1 | 157979849 | T | A | 0.685 | -0.129 | 0.025 | 0.853 | 2.64X10-07 | I | 0.988 |
| rs10437340 | 1 | 157977433 | G | C | 0.685 | -0.129 | 0.025 | 0.853 | 2.64X10-07 | G | - |
| rs4656784 | 1 | 157593504 | A | G | 0.75 | 0.144 | 0.028 | 0.922 | 2.69X10-07 | I | 0.839 |
| rs4656236 | 1 | 157677599 | G | A | 0.725 | 0.135 | 0.026 | 0.859 | 2.80X10-07 | G | - |
| rs4083420 | 1 | 157461462 | A | G | 0.518 | -0.165 | 0.033 | 1.625 | 3.60X10-07 | G | - |
| rs1891187 | 1 | 157895677 | T | A | 0.538 | -0.107 | 0.021 | 0.677 | 3.75X10-07 | G | - |
| rs3116656 | 1 | 157958996 | A | G | 0.683 | -0.129 | 0.025 | 0.851 | 4.08X10-07 | I | 0.943 |
| rs2794526 | 1 | 157892102 | G | A | 0.535 | -0.117 | 0.023 | 0.817 | 5.61X10-07 | I | 0.909 |
| rs12034864 | 1 | 157451604 | A | G | 0.594 | -0.121 | 0.024 | 0.846 | 6.95X10-07 | I | 0.904 |
| rs3027063 | 1 | 157453405 | C | T | 0.596 | -0.120 | 0.024 | 0.830 | 7.42X10-07 | G | - |
| rs3116653 | 1 | 157963534 | G | C | 0.702 | -0.126 | 0.025 | 0.787 | 7.66X10-07 | I | 0.947 |
| rs10751037 | 11 | 80414077 | G | C | 0.51 | 0.164 | 0.033 | 1.603 | 9.07X10-07 | G | - |
| rs3027056 | 1 | 157452855 | G | A | 0.594 | -0.119 | 0.024 | 0.816 | 9.45X10-07 | I | 0.942 |
