# Supplementary material for: A Genome-Wide Association Scan on the Levels of Markers of Inflammation in Sardinians Reveals Associations That Underpin Its Complex Regulation
Source: PLoS Genet. 2012 Jan 26;8(1):e1002480. doi: 10.1371/journal.pgen.1002480 (PMC3266885; doi:10.1371/journal.pgen.1002480)
Supplement: Table S2 — Descriptive statistics for the SardiNIA cohort. The table shows the basic clinical characteristics of the SardiNIA samples. (DOCX) [file pgen.1002480.s005.docx]

**Table S2. Descriptive statistics for the SardiNIA cohort.**

| **Characteristics** | **Value** |
| --- | --- |
| N males/N females | 2579/3447 |
| Age - *mean (min-max)* | 43.6 (14 - 101.3) |
| BMI - *mean (min-max)* | 25.4 (13.9 - 53.3) |
| Smokers *(N)* | 1216 |
| IL-6 (pg/L)- *mean (min-max)* | 3.18 (0.1 – 41) |
| ESR (mm/h) - *mean (min-max)* | 8.00 (1 – 110) |
| MCP-1 (pg/ml)*- mean (min-max)* | 254 (2 – 6080) |
| hsCRP (ng/ml) *- mean (min-max)* | 2.76 (0.15 – 119) |
