# Supplementary material for: A Genome-Wide Association Scan on the Levels of Markers of Inflammation in Sardinians Reveals Associations That Underpin Its Complex Regulation
Source: PLoS Genet. 2012 Jan 26;8(1):e1002480. doi: 10.1371/journal.pgen.1002480 (PMC3266885; doi:10.1371/journal.pgen.1002480)
Supplement: Table S3 — Quality Control data for Affymetrix 6.0 chips and imputation. (DOCX) [file pgen.1002480.s006.docx]

**Table S3.** **Quality Control data for Affymetrix 6.0 chips and imputation.**

| ***Quality checks for* *Affymetrix 6.0 markers*** | ***# Markers filtered*** |
| --- | --- |
| all | 934,968 |
| call rate < 95% | 28,063 |
| MAF < 1% | 150,931 |
| >12 mendelian errors | 2,160 |
| >2 errors on duplicate Ids | 161 |
| >3 errors with 500K genotypes | 3,071 |
|  |  |
| *Affymetrix6.0 QCed autosomal markers* | *727,541* |
| *Affymetrix 6.0 QCed chrX markers* | *27,914* |
| *Affymetrix 6.0 QCed markers* | *755,455* |
|  |  |
| ***Total QCed markers after merging Affymetrix chip genotypes*** |  |
| *autosomal markers* | *731,209* |
| *chrX markers* | *28,004* |
| *Total markers* | *759,213* |
|  |  |
|  |  |
| **Imputed Markers QC(HapMap II)** | **# Markers** |
| All autosomal markers (genotyped and imputed) | 2,659,112 |
| *Imputed* | 1,899,899 |
| rsqr ≤ 0.3 | 691,48 |
| Monomorphic imputed | 132 |
| Excluded for Bad inheritance | 235,847 |
|  |  |
| *Total QCed markers (imputed only)* | 1,594,772 |
| *Total QCed markers (imputed and genotyped)* | 2,353,985 |
