# Supplementary material for: A Genome-Wide Association Scan on the Levels of Markers of Inflammation in Sardinians Reveals Associations That Underpin Its Complex Regulation
Source: PLoS Genet. 2012 Jan 26;8(1):e1002480. doi: 10.1371/journal.pgen.1002480 (PMC3266885; doi:10.1371/journal.pgen.1002480)
Supplement: Table S4 — Quality Controls for the MetaboChip and ImmunoChip data-sets. The table shows the breakdown of each criteria applied and the relative number of markers removed. The same marker could have failed one or more criteria. a excess was defined as >1% of the families. b SNPs on chromosomes X and Y were discarded for the analysis with EMMAX. (DOCX) [file pgen.1002480.s007.docx]

**Table S4. Quality Controls for MetaboChip and ImmunoChip** **custom arrays.**

| **Filters** | **#Markers (MetaboChip)** | **#Markers (ImmunoChip)** |
| --- | --- | --- |
| None | 196,726 | 196,524 |
|  |  |  |
| Call rate <98% | 13,830 | 10,776 |
| MAF = 0 | 38,565 | 31,038 |
| HWE pvalue < 10-6 | 576 | 1,397 |
| Excess MI ^a^ | 951 | 1,742 |
| chrX and chrY^b^ | 105 (93 + 21) | 1,153 (767+386) |
| Insertions/deletions | --- | 718 |
| Build 37 mapping | 888 | 18 |
|  |  |  |
| *Total QCed autosomal Markers:* | 142,790 | 151,085 |
